# Overview of the Most Promising Radionuclides for Targeted Alpha Therapy: The “Hopeful Eight”

**DOI:** 10.3390/pharmaceutics13060906

**Published:** 2021-06-18

**Authors:** Romain Eychenne, Michel Chérel, Férid Haddad, François Guérard, Jean-François Gestin

**Affiliations:** 1 Groupement d’Intérêt Public ARRONAX, 1 Rue Aronnax, F-44817 Saint-Herblain, France; Francehaddad@subatech.in2p3.fr; 2 Université de Nantes, Inserm, CNRS, Centre de Recherche en Cancérologie et Immunologie Nantes—Angers (CRCINA)—UMR 1232, ERL 6001, F-44000 Nantes, France; michel.cherel@univ-nantes.fr (M.C.); francois.guerard@univ-nantes.fr (F.G.); 3 Laboratoire Subatech, UMR 6457, Université de Nantes, IMT Atlantique, CNRS, Subatech, F-44000 Nantes, France

**Keywords:** targeted alpha therapy, α-emitters, actinium-225, astatine-211, bismuth-212, bismuth-213, lead-212, radium-223, terbium-149, thorium-227

## Abstract

Among all existing radionuclides, only a few are of interest for therapeutic applications and more specifically for targeted alpha therapy (TAT). From this selection, actinium-225, astatine-211, bismuth-212, bismuth-213, lead-212, radium-223, terbium-149 and thorium-227 are considered as the most suitable. Despite common general features, they all have their own physical characteristics that make them singular and so promising for TAT. These radionuclides were largely studied over the last two decades, leading to a better knowledge of their production process and chemical behavior, allowing for an increasing number of biological evaluations. The aim of this review is to summarize the main properties of these eight chosen radionuclides. An overview from their availability to the resulting clinical studies, by way of chemical design and preclinical studies is discussed.

## 1. Introduction

Nuclear medicine is a discipline whose applications can have two different purposes: imaging, with visualization of a radionuclide distribution in the organism, or therapy, with specific irradiation of malignant cells. Whatever the intended application, it is mainly based on the administration of drugs defined as radiopharmaceuticals. These radioactive tracers are usually made up of: a radioactive moiety (a unit involving a radionuclide) whose radiations allow localization (γ or β^+^ emitters) or destruction of targeted cells (α, β^−^ or electron Auger emitters); and a molecule to carry it to the target. Unlike imaging that uses radionuclides emitting highly penetrating radiations, radiotherapy favors those that strongly interact with matter leading to low penetration. In that case, α, β^−^ or Auger electron emitters are used to lead to death of malignant cells. This radiopharmaceutical design is at the center of the general concept of targeted radionuclide therapy (TRT) gathering several promising methods such as peptide receptor radionuclide therapy (PRRT) or radioimmunotherapy (RIT) using more specifically β-emitters or α-emitters [1]. The nature of the emitted particle is essential because it has an influence on the induced biological effect. This difference is illustrated by two main features of emitted particle: the path length and the linear energy transfer (LET) [2]. From a biological point of view, irradiation of cells results in direct (by energy transfer, as DNA damage and cross-fire effect) and indirect cellular mechanisms (reactive oxygen species (ROS) generated by water radiolysis and radiation-induced bystander effect (RIBE) described as the spread of signals from irradiated to neighboring cells inducing apoptosis of cells that are not directly exposed to ionizing radiations or an immune response known as abscopal effect) [3,4]. All these possible biological damages result in a relative biological effectiveness (RBE), an essential factor for dosimetry calculations necessary to estimate radiobiological effects. RBE is defined as the ratio of the doses required by two radiations to achieve the same biological response. This value is often calculated from a standard dose, usually of external beam radiation, in relation to other ionizing particles. Consequently, the RBE depends on several parameters such as the particle type, absorbed dose or tissue type, and allows to compare two modalities and better estimate the therapeutic potential for the same application [5].

The low LET (0.2 keV.µm^−1^) of β^−^ emitters reflects the low amount of energy transferred per unit of distance from the ionizing radiations to the targeted tissues, mainly resulting in DNA single strain breaks with a higher probability for the cell to repair the damages. Consequently, a high number of β^−^ particles is necessary in order to saturate cellular repair mechanism or to cause multiple DNA single strain breaks that will have the same effect as DNA double strain breaks (DSBs) and induce cell death. In addition to this direct ionization, irradiation with β^−^ particles has also indirect effects with the increase of intracellular ROS concentration (oxidative stress) that have also an impact on biomolecules such as lipids, proteins and especially DNA. Moreover, the long path length (1–10 mm) of the emitted electron provides the advantage of allowing to remotely irradiate non-targeted malignant cells especially in large tumor mass. This phenomenon is named crossfire effect. The mechanism of action demonstrated a real interest for macroscopic or heterogeneous tumors even if the particle can also be released out of the tumor and potentially reach healthy tissues. This method is not adapted for microscopic or disseminated cell clusters [2,5]. α-emitters, with high LET (about 100 keV.µm^−1^) and a path length around 50–100 µm, have a high ionization potential associated to high energy (2–10 MeV). The mass and charge (+2) of the emitted ^4^He nucleus directly impact DNA, leading to a significantly higher DSBs probability than β-emitters, inducing a higher cytotoxicity. Besides, this short path length also reduces irradiation of surrounding healthy cells, which can been seen as an advantage in terms of treatment toxicity [5,6]. These specificities made them efficient for the treatment of small tumors or disseminated metastases and isolated cancer cells [7,8,9]. Among all the existing α-emitters, only few have suitable properties for a potential clinical application. Availability as well as short half-lives of some radionuclides of interest were often an obstacle to targeted alpha therapy (TAT) development. However, for a few years, a better general knowledge of these emitters, especially regarding production processes, radiochemistry or even new biomarkers, has resulted in a growing interest for therapy using α-emitters.

Several reviews have already reported the advances as well as the global trends on α-emitters which were considered at that time [10,11,12,13]. In addition to the update, this review gives a wider overview of most promising α-emitters from their production pathways and availability to the latest clinical studies, by way of (radio) chemical design and preclinical investigations. The main idea is to provide as many elements as possible in order to evaluate the potential interest of these α-emitters in order to better understand the future of TAT.

## 2. Actinium-225

**Figure 1:**
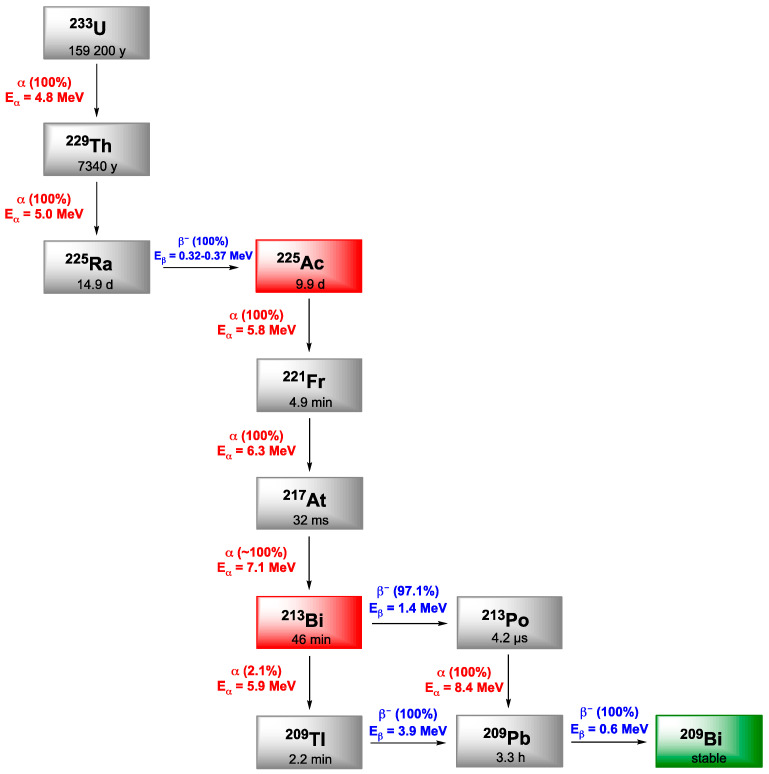
Decay schemes for the production of ^225^Ac and ^213^Bi.

Actinium is the first element of the actinide series, and among its 32 known isotopes, only ^228^Ac and ^227^Ac occur naturally as part of the decay chains of ^232^Th and ^235^U, respectively. ^227^Ac is the most abundant isotope, exhibiting a long half-life of 21.7 years and decaying mainly through β^−^ emissions. ^228^Ac is also a β^−^ emitter and is extremely rare. ^225^Ac is part of the ^237^Np decay chain which has disappeared in Nature but was recreated artificially. ^225^Ac exhibits features that have made it a promising candidate in nuclear medicine applications. It has a half-life of 9.9 days. Its disintegration follows a six-step decay chain to reach stable nuclei, that generates multiple alpha particles contributing to increasing its potential cytotoxicity in comparison with other α-emitters (Figure 1). Besides, with the γ-emission of some of its daughter nuclides such as ^221^Fr or ^213^Bi, it provides the possibility to trace it after injection. Nevertheless, it has to be mentioned that these radiations make the reaction monitoring difficult and the secular equilibrium has to be reached before one can measure a reliable radiochemical yield (at least 6 h).

### 2.1. Production

^225^Ac can be recovered from the long decay process of ^233^U (159,200 years), ^229^Th (7340 years) and ^225^Ra (14.9 days). Given the very long half-life of these isotopes, ^225^Ac can only be obtained in intervals of 6 to 8 weeks and with regular purification processes (ion exchange chromatography columns) in order to remove other daughter nuclides. Currently, the demand in ^225^Ac is met by ^229^Th systems from ^233^U produced in the 1960s and early 1970s (by neutron irradiation of ^232^Th) in nuclear research programs, especially as weapon material or power reactor fuel (mainly in the US). However, at the end of the 1970s, the thorium fuel cycle was abandoned in favor of plutonium-fueled fast reactors and an important stockpile of ^233^U was stored. It was only recently that it has been possible to use this excess of ^233^U in order to supply ^225^Ac and ^213^Bi for medical applications, but, with the increasing interest in these nuclides, this method alone will not be able to satisfy the growing demand. To overcome availability issues, new production routes have been developed. In addition to direct production routes, ^225^Ac can also be isolated after decay of its three parent isotopes ^233^U, ^229^Th and ^225^Ra. Overall, these alternatives are divided into three general methods: production of ^229^Th, production of ^225^Ac and ^225^Ra from ^226^Ra or production of ^225^Ac and ^225^Ra from irradiation of ^232^Th or uranium [14,15,16].

Among them, some production routes turned out to be interesting for the large-scale development of ^225^Ac. First, with direct production of ^229^Th, through the exposure of ^226^Ra, ^228^Ra or ^227^Ac targets to intense fluxes of thermal neutrons. Even if they all showed the possibility to generate ^229^Th, co-production of ^228^Th (difficult to separate from ^229^Th) from ^226^Ra and ^227^Ac targets as well as the low yield obtained from ^228^Ra targets are limitations that have to be overcome for a consequent contribution in the ^225^Ac production. We should also note that the long half-life of ^229^Th is a parameter that is not supportive of this method [17,18]. Secondly, proton irradiation of ^226^Ra targets via the ^226^Ra(p,2n)^225^Ac nuclear reaction in a low-energy cyclotron is one of the most promising routes. With a proton energy beam around 16 MeV, this method showed high production yields from a simple procedure and with high purity, without production of long-lived actinium isotopes. Nevertheless, some disadvantages include the lack of routine experience with ^226^Ra targets and the need to handle ^222^Rn daugther, a radioactive gas, with a half-life of 3.82 d [19,20]. This also holds true for the photoproduction route, a promising alternative that uses ^226^Ra target irradiated with gamma rays produced by bremsstrahlung [21]. Finally, another method that has also received attention is the irradiation of ^238^U or ^232^Th targets [22,23,24] with high energy protons (spallation of ^238^U or ^232^Th with >100 MeV proton bombardment) following ^238^U(p,x)^225^Ac or ^232^Th(p,x)^225^Ac reactions. While this route allows the production of several GBq of ^225^Ac with large accelerators, the spallation reaction of ^232^Th is often preferred, especially due to the easier handling and absence of fissile elements co-production. Multiple chemical purification steps are necessary to remove impurities mainly due to co-production of radionuclides, while the simultaneous co-production of ^227^Ac is problematic as its half-life is 21.7 y. Even if the presence of ^227^Ac was identified, it is estimated that the low ratio (0.1–0.2% of the relative activity of ^225^Ac) do not impact dosimetry although this remains to be confirmed by toxicity studies before clinical use. However, waste management remains a major concern due to ^227^Ac long half-life and will require strategies with potentially elevated associated costs. Interestingly, additional purification processes such as isotope separation (Isotope Separation On-Line, ISOL at TRIUMF) or a production route from ^225^Ra generated after proton irradiation of ^232^Th, have emerged recently, allowing a decrease in ^227^Ac content and thus the recovery of ^225^Ac with higher purity [25,26].

Whether by ^233^U decay or by one of the other production routes mentioned above, sources of ^229^Th as generators is the main process for providing ^225^Ac, representing currently more than 95% of worldwide production. More precisely, after extraction of ^229^Th and dissolution in 8 M HNO_3_, a first step using anion exchange resins such as MP1, AG1 × 8 or Dowex 1 × 8 allows the separation of mixtures of ^225^Ac and ^225^Ra. Isolation of ^225^Ac is then performed by a second step, either with a cation exchange column [27], solid phase extraction chromatography [17] or a combination of anion and cation exchange resins [28]. These strategies result in the recovery of ^225^Ac in high yields (80–95%) and with high radionuclidic purity for a use in preclinical and clinical trials.

Currently, ^229^Th generator systems are the only method to provide this radionuclide on a routine basis. General interest in ^225^Ac is continuously increasing to the point that it is estimated that the worldwide demand will be multiplied by at least two or three each coming year. Existing ^233^U stocks are being made available by the US Department of Energy to the TerraPower company (Bellevue, WA, USA) in order to further increase the availability of generator-based ^225^Ac. Even if other interesting options for ^225^Ac production appeared, further work as well as consequent investment in infrastructure and equipment will be necessary before scaled-up production is observed.

### 2.2. Radiolabeling Chemistry

All actinium isotopes are unstable and available in limited amounts, which negatively impacts any progress in the study of this element’s coordination chemistry. However, with a [Rn] 6d^1^ 7s^2^ electronic configuration, actinium is commonly found in aqueous solution in the (+III) oxidation state like other actinides or rare earth elements. These trivalent ions are highly charged entities that interact only in an electrostatic manner, resulting in hard acceptor properties according to Pearson’s hard and soft acids and bases (HSAB) theory. Therefore, for an efficient actinium complexation, the ligand has to contain hard coordination sites such as oxygen or nitrogen Lewis bases. For the same reasons, the coordination number is ruled by the size of the corresponding cation or anion. Ac(III) is described as having a large cationic radius of 1.12 Å and thus to be more adapted to polydentate ligands with high denticity from 8 up to 12.

The macrocycle 1,4,7,10-tetraazacyclododecane-1,4,7,10-tetraacetic acid (DOTA) has amply demonstrated its efficiency for complexation of many metallic trivalent ions. Well-known in terms of reaction conditions but also by regulatory authorities, it was naturally studied for ^225^Ac chelation. In addition to a fast clearance from blood, the biodistribution profile of ^225^Ac-DOTA complex showed a low uptake in liver and bones, target organs of free ^225^Ac, which validated a promising in vivo stability at the preclinical stage [29]. These encouraging first results led to the development of functionalized DOTA derivatives such as *p*-SCN-Bn-DOTA or MeO-DOTA-NCS, allowing conjugation to biomolecules via the lysine residues of antibodies. However, the reaction conditions required for quantitative complexation of ^225^Ac (50–60 °C for 30–60 min) is not compatible with proteins which has resulted in the development of a two-step radiolabeling procedure [30]. Although this method showed a clear reliability and has been reported for the labeling of several antibodies, low overall radiochemical yields (RCYs) attributed to a degradation of the isothiocyanate function after the heating step were noticed. Consequently, a more specific two-step method based on Michael addition of a maleimide derivative on cysteine residues emerged, allowing a gain in efficiency [31]. In comparison to these procedures, a real step forward was made later with the report of a direct labeling approach with improved RCYs and specific activity but also that is applicable under milder reaction conditions [32]. However, despite many advantages, this method provided a grafting more than 10 chelators per antibody that can be problematic as it could affect the immunoreactivity of some modified antibodies. It has to be noticed that another two-step procedure based on an inverse electron demand Diels-Alder (IEDDA) click reaction between a DOTA-tetrazine derivative and a *trans*-cyclooctene antibody conjugate was recently described [33]. In addition to a very short reaction time, promising RCYs and in vivo stability, the potential use in classic RIT and especially in pretargeted α-radioimmunotherapy (PRIT) was clearly demonstrated and could open new prospects [34].

Nevertheless, some studies have raised questions about DOTA-actinium complex’s thermodynamic stability attributed to the chelating cavity of DOTA macrocycle that may not be adapted to the large size of ^225^Ac(III) ion resulting in potential transmetalation with other ions [29,30]. In vivo evaluations of other possible ligands such as acyclic derivatives (ethylenediaminetetraacetic acid, EDTA or *trans*-cyclohexyl diethylenetriaminepentaacetic acid, CHX-A’’-DTPA) were not good options as they demonstrated a lack of kinetic inertness [29,35]. Despite promising stability of complex formed with HEHA macrocycle (1,4,7,10,13,16-hexaazacyclohexadecane-*N,N′,N″,N‴,N″″,N″″′*-hexaacetic acid), results were disappointing after functionalization (HEHA-NCS) [36,37].

**Figure 2:**
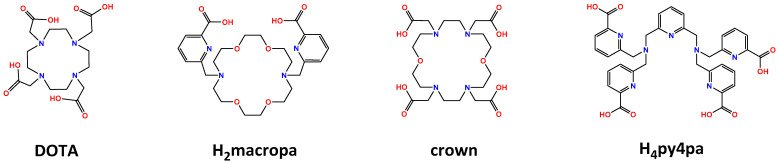
Chemical structures of main ^225^Ac chelators.

New chelating agents based on structures bearing picolinic acid arms [25,38,39] including aza-crown-ether macrocycles [40,41] have recently emerged for complexation of ^225^Ac (Figure 2). Among all the reported chelators, the 18-membered macrocycle macropa [40], the new “crown” aza-macrocycle [41] and the undecadentate pyridyl-based H_4_py4pa [39] showed an efficient complexation at room temperature as well as an interesting stability against competing ions or in serum. After conjugation to a peptide or an antibody of interest, they all showed a promising in vivo stability highlighted by a high uptake in tumor and a low accumulation in other organs such as liver or bones.

### 2.3. Preclinical Studies

Beyond the stability brought by the chelating unit towards ^225^Ac, an aspect that has to be taken into consideration is the outcome of ^225^Ac daughters after in vivo administration. As mentioned before, ^225^Ac generates several α particles that raise concerns. Indeed, in addition to potential irradiation of non-targeted tissues, release of these daughter nuclides in vivo can result in unwanted toxicity [42]. Generally, daughters are released from the chelating agent and pass into the bloodstream before it accumulates in the targeted organs. Daughter nuclides of ^225^Ac that have sufficient half-life and decay by alpha emission are ^231^Fr and ^213^Bi that were reported to have affinity for kidneys and possibly lead to renal toxicity [43]. Nevertheless, this recoil phenomenon can be limited by a fast internalization in the targeted cells or a local administration of ^225^Ac-radioconjugate or by sequestration of the nuclide in nanoparticles. This cascade decay was at the origin of the concept of atomic in vivo nanogenerator for TAT [44].

Production routes as well as chemistry aspects previously presented are detailed in two complete and recent reviews dedicated to ^225^Ac [15,45]. Section 2.2 clearly illustrated that DOTA macrocycle appears as the better compromise for ^225^Ac chelation as evidenced by the fact that it is the only chelating agent used in current clinical trials. Nonetheless, a wide range of chelators were developed for ^225^Ac and even if only a few have demonstrated appropriate features for a potential use in TAT, many preclinical evaluations were reported. A non-exhaustive list of studies published in the past five years is presented in Table 1. The aim of this summary is to give a detailed overview of the recent advances with ^225^Ac mentioning the pathology model as well as the target studied, the chelating agent selected and the level of investigations. However, much more attention was given to ^225^Ac-immunoconjugates that succeed to be evaluated in clinical phase (see next section).

### 2.4. Clinical Evaluation

Acute myeloid leukemia (AML) is a hematologic malignancy characterized by an uncontrolled proliferation of abnormal myeloid blasts unable to differentiate into healthy mature cells. Unlike normal cells, myeloid cells are characterized by the expression of specific targets such as CD33, a sialic acid transmembrane receptor, and CD45, a protein tyrosine phosphatase, both involved in modulation of immune cell functions [9]. Lintuzumab (or HuM195) is a humanized mAb that has emerged as a promising vector for targeting CD33 with high affinity and without specific immunogenicity. First reported with ^213^Bi, investigations of lintuzumab labeled with ^225^Ac started with a study in cynomolgus monkeys in order to evaluate pharmacokinetics and toxicity of the radioimmunoconjugate. No significant toxicity was noticed after administration of 28 kBq/kg while cumulative doses in the 215–370 kBq/kg range revealed signs of impact on hematologic and renal functions (mainly related to redistribution of daughter nuclides) [63]. This work was followed by the first clinical investigation against AML through a dose escalation study. 18 patients received a single administration of ^225^Ac-lintuzumab at doses of 18.5, 37, 74, 111 and 148 kBq/kg. Even if a clear reduction of marrow blasts was noticed for all doses, more or less pronounced myelosuppression was reported as most common toxicity symptom, and the maximum tolerated dose was determined to be 111 kBq/kg [64]. From these first results, investigations were pursued in a phase II trial in patients with untreated AML and treated with two administrations of 55 or 74 kBq/kg [65]. Other phase I dose-escalation were also performed from fractionated doses of ^225^Ac-lintuzumab in combination with low-dose cytarabine (a chemotherapy agent for inhibition of cells proliferation by interaction with DNA) [66] or associated with venetoclax, a BCL-2 inhibitor inducing cells apoptosis [67] (NCT03867682).

Prostate-specific membrane antigen (PSMA) is a type II membrane glycoprotein highly expressed in prostate cancer cells. This promising target is involved in a large majority of ^225^Ac investigations, especially with ^225^Ac-PSMA-617. This radioconjugate is based on a glutamate-urea-lysine sequence known as a PSMA inhibitor (with internalization), a naphtylic linker to favor tumor uptake as well as renal clearance, and DOTA as chelating agent [68,69]. Several clinical evaluations in advanced stages showed that treatment cycles with adapted administered doses (8 to 4 MBq), resulted in a significant therapeutic effect highlighted by a large decrease in tumor evolution markers (prostate-specific antigen (PSA), alkaline phosphatase) and appeared as the best balance between possible toxicity and tumor response [70]. More details about the work reported with ^225^Ac-PSMA-617 can be found in Kratochwil’s recent review [71], the therapeutic potential of this treatment is real and further evaluation, especially a phase I dose escalation combined with DNA damage repair inhibitors is still ongoing (NCT04597411) [72]. It has to be mentioned that a similar radioligand, ^225^Ac-PSMA-I&T, was recently tested in clinical applications in the same model, in patients after ^177^Lu-PSMA-617 treatment failure [73,74].

An alternative to the potential limitations encountered with radioligand therapy would be to evaluate ^225^Ac-labeled Ab. For this reason, J591, a mAb well known to bind with high affinity an extracellular domain of PSMA that is different of PSMA-617 moiety, is interesting. ^225^Ac-J591 is currently studied in a phase I dose-escalation in order to determine the dose-limiting toxicity as well as the maximum tolerated dose [75] (NCT03276572). Two other clinical trials are in the recruiting phase with the aim to evaluate the possibility of treatment with multiple or fractionated dose of ^225^Ac-J591 (NCT04576871, NCT04506567). PSMA has been widely targeted with ^225^Ac or others α emitters such as ^213^Bi or ^227^Th. A very recent review provides a detailed overview of this work [76].

Finally, PSMA is not the only target to be investigated for treatment of prostate cancer and other pathways upregulated by androgen receptors are also appropriate. These receptors were described to be involved in regulation of genes responsible for DNA damage repair mechanisms that could possibly result in resistance of cancer cells. Human kallikrein peptidase 2 (hK2), a specific enzyme structurally quite similar to PSA and whose expression is governed by androgen receptor, emerged as a specific and promising marker [77]. The specific monoclonal antibody (mAb) hu11B6 radiolabeled with ^225^Ac was recently reported in an interesting mechanism of action towards hK2. Indeed, due to internalization of ^225^Ac-hu11B6 after binding to hK2, α radiations induce DNA damage to cancer cells, resulting in activation of androgen receptor for DNA repair [78]. It also leads to the activation of KLK2, gene coding for hK2 production, resulting in enzyme production. Therefore, the increase in hK2 levels favors cells targeting by ^225^Ac-hu11B6 and this phenomenon was highlighted in in vivo experiments with an increasing uptake in tumors over time [79]. This strategy is to object of a clinical evaluation, recruitment started for a phase I study in order to validate the promising preclinical results in patients (NCT04644770).

Neuroendocrine tumors (NETs) are characterized by an abnormal proliferation of hormone producing cells that can possibly develop in privileged sites such as gastrointestinal tract, pancreas or lungs. These tumors are well known to overexpress somatostatin receptors in comparison with healthy tissues, which represents an interesting biomarker for peptide receptor radionuclide therapy. In the case of ^225^Ac, it started with ^225^Ac-DOTATOC evaluated in a rat pancreas NETs (AR42J cells) model. Activities up to 20 kBq showed a significant effect on tumor growth with only negligible long-term toxicity on kidneys when higher dose (30–125 kBq) also had tumor effect but were associated to histopathological changes in kidneys. In comparison with ^177^Lu-DOTATOC (doses of 450 kBq and 1 MBq), even if no toxicity was observed, treatment efficacy was clearly lower [80]. The first clinical study in 34 patients with progressive NET validated the results observed in preclinical phase and allowed to determine that, for a single cycle, the maximum tolerated dose was of 40 MBq. Despite positive conclusions and good tolerability, further investigations are necessary to clarify the treatment protocol [81]. ^225^Ac-DOTATATE, an analogue compound, was also recently studied in patients with stable or progressive disease after treatment with ^177^Lu-DOTATATE [82].

Substance P (SP) is a neuropeptide known as a natural ligand of transmembrane neurokinin type-1 receptor (NK-1) overexpressed in glioblastoma multiforme (GMB). ^225^Ac-DOTA-SP was evaluated in human glioblastoma cell lines and demonstrated promising effects on cell viability by induction of apoptosis [83]. A clinical phase is ongoing in patients with glioma, and first results showed a positive tumor response after intratumoral administration [84].

Finally, based on similar approaches than ^225^Ac-radioimunoconjugates presented above, ^225^Ac-FPI-1434 is under investigation in a phase I trial with the aim to determine tolerability, pharmacokinetic and treatment efficacy in patients with advanced solid tumors. This therapeutic agent is based on a humanized mAb (AVE1642) with recognition properties to type I insulin-like growth factor receptor (IGF-1R), a tyrosine kinase receptor overexpressed in solid tumors such as breast, prostate or non-small lung cancer (NCT03746431).

## 3. Astatine-211

**Figure 3:**
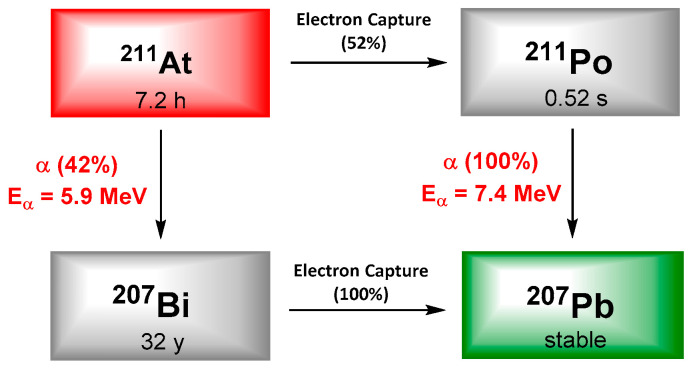
Simplified decay scheme of ^211^At.

Astatine is often reported as the rarest natural element on Earth, and exhibits 32 isotopes, none of them being stable. Even if most of these isotopes are α-emitters, only ^211^At exhibits suitable physical properties for TRT. It decays with a half-life of 7.21 h and following a dual branch process resulting in the emission of 5.9 and 7.5 MeV α particles as well as X-rays (77–92 keV). Whatever the branch, one alpha particle is emitted, qualifying ^211^At as a 100% α-emitter. It is interesting to notice that potential radiotoxicity of ^211^At daughters’ is negligible; especially because of the short half-life of ^211^Po and the very low amount of ^207^Bi generated. Finally, the emission of X-rays in the adapted energy range of common γ-detectors, offers the possibility of monitoring by single photon emission computed tomography (SPECT) imaging (Figure 3).

### 3.1. Production

This radionuclide is cyclotron-produced by α particle bombardment of a natural ^209^Bi target following the ^209^Bi(α,2n)^211^At nuclear reaction which reaches a maximum cross-section at 31 MeV [85,86]. The α particles beam should have an energy between 21 and 29 MeV. Beyond 29 MeV, the co-production of ^210^At (8.1 h) by the ^209^Bi(α,3n)^210^At reaction makes difficult any use in pharmaceutical applications. Indeed co-production of ^210^At has to be avoided because it decays almost exclusively to ^210^Po (138.4 days), well known to be highly toxic [87]. Few cyclotrons in the world are currently able to accelerate α-beams to energies higher than 25 MeV, consequently, the availability of ^211^At is limited to a few nuclear medicine centers [88]. In addition, its half-life does not allow for long distance delivery, which implies that a network of producers must be set up to support the development of this radionuclide. Nevertheless, the production technology is under control and the capacity of production can be largely increased by using old cyclotrons or new ones proposed by IBA (30Xp, Julich, Polatom) or Sumitomo (several centers in Japan). Another production route mainly based on electron capture of ^211^Ra via a ^211^Rn/^211^At generator are being developed. The main interest of this method is to allow an increase in ^211^At availability by widening its distribution through the longer half-life of ^211^Rn (14.6 h), but also to avoid some production issues associated with the current method such as the contamination with ^210^At and ^210^Po. Despite the reported proof of concept, this alternative showed limitations, especially because of the specific and uncommon techniques required to produce ^211^Rn (heavy ion irradiation of ^209^Bi target by a ^7^Li beam), difficulties for the isolation of ^211^At from co-produced ^207^At and ^207^Po, as well as a moderate activity of ^211^At obtained at the end of the process [89].

After production, the irradiated target contains several isotopes such as ^209^Bi, ^210^At, ^210^Po and the expected ^211^At. A purification process is necessary to remove impurities and reach the highest ^211^At chemical and radiochemical purity. However, if ^209^Bi or ^210^Po residues can be eliminated by this step, it is not possible to separate ^210^At from ^211^At that is why the incident energy of the projectile is limited to 29 MeV in order to avoid ^210^At production. Two main purification methods were developed: (i) by dry distillation: the target is heated beyond the boiling point of ^211^At (337 °C) that is largely different from of the other radionuclides (1564 °C for ^209^Bi and 962 °C for ^210^Po). Vaporized ^211^At is carried over by a flow of nitrogen and condensed in the chosen medium [90,91]; and (ii) by wet extraction: after dissolution of the target in concentrated nitric acid, the acid phase is removed by evaporation and the dry residue containing ^209^Bi and ^211^At is dissolved in diluted nitric acid or hydrochloric acid. Pure ^211^At is then recovered by extraction with diisopropyl ether [92]. Both methods are reliable, resulting in high recovery yields (around 80–90%), with a duration of 20–30 min and about 1 h, respectively. However, despite the advantages of wet extraction (simple process, cheap equipment), the number of steps that require manipulation of the radioactivity during the process has long been a limitation with high activity production. Nevertheless, even if an automated process was recently developed for wet extraction, dry distillation is still widely used for purification of ^211^At. It is important to note that alternative methods based on the wet chemistry route were recently reported for purification and isolation of ^211^At, as purification on a tellurium-packed column [93], a solid-state support inert polymer resin [94] or strong anion exchange SPE spin column [95] or extraction with organic solvents such as 3-octanone or methyl isobutyl ketone [96].

### 3.2. Radiolabeling Chemistry

Given that no stable isotopes of astatine exist and that its availability is quite limited, some of the physicochemical properties of this element were extrapolated by analogy with iodine or predicted using theoretical methods. However, the supplementary 5f electrons of At imply that relativistic effects and especially spin-orbit coupling must be considered, impacting significantly its electronic properties and reactivity such as polarizability or electronegativity [97]. Therefore, even if similarities with iodine were clearly identified, striking differences in its chemical behavior were reported too, making it important to consider the oxidation state of At species. If some analogy with iodine was confirmed by the reactivity of the (−I) oxidation state, At^−^ (one of the most stable form, obtained in reducing conditions), some differences have been observed with positive oxidation states (+I or +III obtained in oxidizing conditions), especially with the observation of metal-like properties [98,99]. Despite its clearly demonstrated metallic character, covalent chemistry is largely preferred for radiolabeling. From all these observations, reactions described for the preparation of astatinated molecules are mainly based on iodine radiochemistry. Besides, astatine electronic properties make the C-At bond weaker than others C-halogen bonds. (Hetero)aryl-At bonds are almost exclusively used as they are the only compounds to exhibit sufficient stability for use in vivo. Generally, labeling methods reported in the literature for radioiodination of organic compounds, often based on basic aromatic electrophilic or nucleophilic substitutions (SE_Ar_ or SN_Ar_), remain valid for astatination. Consequently, this radiolabeling chemistry of ^211^At can be divided into two general approaches, the electrophilic one based on the At^+^ form and the nucleophilic one using the At^−^ form. The At^+^ form is generated using usual oxidizing agent such as chloramines or N-halosuccinimide derivatives, but difficulties to control this species may be encountered as overoxidation to At(+III) is complicated to prevent. Classic SE_Ar_ reaction such as halodeprotonation appeared to be less efficient than with radioiodine [100] and was not adapted for application with proteins [101]. On the other hand, halodemetallation was reported to be more efficient due to the polarization of the carbon-metal bond, favoring the substitution reaction. Although initially developed with organomercuric derivatives [102], trialkylaryltin were preferred, Sn(alkyl)_3_ being good leaving groups associated to a lower toxicity [103]. Recently, silicon derivatives were proposed as possible alternative to stannylated compounds [104]. The other approach is based on the At^−^ species, presented to be easier to handle due to a larger predominance domain than At^+^. This strategy was first tested with halogen exchange reaction, but the excess of non-separable iodinated analogue, resulting in a low specific activity, limits its scope of application [105]. The halodediazotation reaction from arenediazonium salts was also evaluated for astatination, however, the various side products generated made this method anecdotal [106,107]. A new strategy based on hypervalent iodine compounds and aryliodonium salts appeared to be promising for preparation of astatoaryl compounds, especially because of more convenient purification steps in comparison with other techniques [108]. Finally, arylboron derivatives (boronic ester or boronic acid) are also interesting since they can be used following a nucleophilic approach associated with copper catalyst [109].

Alternatively the B-At bonds were reported as exhibiting a higher bond enthalpy than aryl-At bonds. This resulted in the development of boron clusters and especially *closo*-decaborate, which appeared as promising candidate for astatine labeling [110].

### 3.3. Preclinical Studies

**Figure 4:**
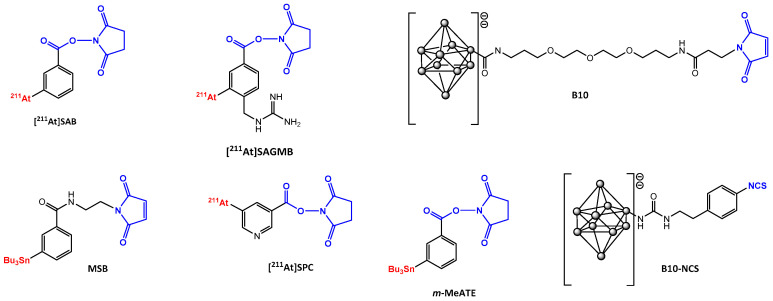
Structures of main prosthetic groups used for ^211^At radiolabeling. [^211^At]SAB: *N*-succinimidyl-3-[^211^At]astatobenzoate [111,113]; [^211^At]SAGMB: *N*-succinimidyl 3-[^211^At]astato-4-guanidinomethyl benzoate [123]; B10: maleimido-*closo*-decaborate(2-) derivative [110]; MSB: *N*-2-(maleimido)ethyl-3-(trimethylstannyl)benzamide [115]; [^211^At]SPC: *N*-succinimidyl 5-[^211^At]astato-3-pyridinecarboxylate [127]; *m*-MeATE: *N*-succinimidyl-3-(trimethylstannyl)-benzoate [114]; B10-NCS: isothiocyanatophenyl-*closo*-decaborate(2-) derivative [110].

Regarding the specific case of protein astatination, two strategies are possible: a two-step approach (via the preparation of radiolabeled prosthetic groups) or a direct labeling of pre-modified protein. Both options require the preparation of a bifunctional precursor bearing a functional group allowing the labeling and a second one for the conjugation with the biomolecule via lysine or cysteine residues (Figure 4). *N*-succinimidyl-3-[^211^At]astatobenzoate ([^211^At]SAB) was largely studied as prosthetic group for conjugation to lysine residues and was also derived with a maleimide function for coupling to cystein. The main reported approaches are the use of aryltrialkylstannane derivatives [111,112], boron clusters [110] and iodonium salts [113] or labeling of pre-modified antibodies with aryltrialkylstannane function [114,115], clickable moieties [116] and boronic acid functionalization [117]. Additional details about astatine chemistry are available in several complete reviews [88,118,119].

The in vitro and in vivo stability of the astatine linkage is crucial for the development of ^211^At-radioconjugates. Indeed, in vivo release of astatine from astatinated biomolecules can induce toxicity to non-targeted tissues. Free astatine (At^−^ form), just like iodine, showed a high accumulation in thyroid and stomach. Unlike iodine, other organs such as spleen and lungs also revealed quite consequent uptake, attributed to At(+I) species formed after in vivo oxidation of the released astatine. First, many investigations of ^211^At-radioconjugates showed a promising in vitro stability but in vivo deastatination was noticed, mainly related to the metabolism of the compound. It appeared that non-internalized or intact mAb were slowly metabolized and consequently less affected by deastatination process, while small molecules or internalized mAb (more rapidly metabolized) were more sensitive to this phenomenon [120]. Stability of ^211^At-derivatives remains a key point to the development of ^211^At, and even today, it is still necessary to find new radiolabeling strategies leading to higher astatine bond strength.

It is especially with the emergence of the [^211^At]SAB prosthetic group that preclinical and clinical work were really favored. Thereafter, plenty of in vitro experiments were performed mainly to evaluate the cytotoxicity and the dosimetry of the radioconjugates developed. It naturally led to a wide range of preclinical model investigations throughout various biomolecules (mainly antibodies, but also antibody derivatives such as F(ab’)_2_, diabodies, nanobodies or even peptides), towards specific molecular targets (HER2, CD45 or CD38) and with several astatine forms (^211^At-immunoconjugates, small molecules or [^211^At]NaAt). We report herein a non-exhaustive list of published preclinical studies involving an identified pathology model in the last five years (Table 2). This summary gives also interesting details about the most recent preclinical studies as the radiolabeling strategy used (direct radiolabeling or via prosthetic groups) as well as which targeting vector was considered.

In the following discussion, we chose to focus on ^211^At-radioimmunoconjugates that translated to a clinical application (see next section). Overall, most of these works resulted in promising uptake in tumors, tumors growth delay and therapeutic efficacy, confirming the interest of ^211^At for TAT.

### 3.4. Clinical Evaluation

Glioblastoma is the most common and aggressive primary brain cancer and remains associated with a very poor clinical prognosis. Despite protocol treatments, tumor recurrence close to the primary site is often observed, with a dramatic effect on recovery. Monoclonal murine antibody 81C6 recognizes the extracellular matrix antigen tenascin overexpressed mainly in gliomas and melanomas. Initially conjugated with ^131^I, the corresponding radioimmunoconjugate demonstrated a real survival benefit in patients after administration in surgery-created resection cavities (SCRC) [144], which naturally led to the transposition with its astatinated analogue. ^211^At-81C6 was first investigated in a therapeutic study, in rats grafted with TE-671 human rhabdomyosarcoma neoplastic meningitis cell line. A specific therapeutic effect was confirmed with a significant prolongation of survival in animals treated with a single injection 440 and 670 kBq, without any sign of toxicity even after 295 days [145]. In order to favor a future clinical application, the chimeric version of 81C6 was then evaluated in another glioma model (D-54 MG human glioma xenografts). Biodistribution, dosimetry and toxicity studies confirmed the interest of ch81C6 over its murine form [146]. Data of the first Phase I clinical trial with astatinated radioimmunoconjugate for the treatment of residual central nervous system tumors (glioblastoma multiforme, anaplastic oligodendroglioma and anaplastic astrocytoma) was published in 2008. Additionally to surgery, radio- or chemotherapy, 18 patients received an injection of ^211^At-ch81C6 with doses from 71 to 347 MBq in SCRC. Significant prolongation of median survival was noticed from 31 weeks with the classic protocol to about 54 weeks with ^211^At-RIT, without any sign of dose-limiting toxicity, meaning that this treatment was well tolerated [147]. Despite this proof-of-concept of clinical application with ^211^At, some limitations appeared, especially related to radiolabeling issues at high activity that hampered the determination of the maximum tolerated dose. Prior to this work, a two-step procedure from [^211^At]SAB precursor was developed for high level preparation of ^211^At-ch81C6 in order to support the clinical need [148]. However, with this level of activity, effects on radiochemical or conjugation yields and immunoreactivity were observed and were attributed to α-particle radiolysis. A better comprehension of this phenomenon resulted in the development of an optimized procedure modifying some parameters such as the stannylated precursor (*N*-succinimidyl-3-trimethylstannylbenzoate vs. *N*-succinimidyl-3-tributylstannylbenzoate) or the nature of the solvent used after distillation (MeOH with *N*-chlorosuccinimide vs. CHCl_3_) [149].

Ovarian cancer is often diagnosed late, when extensive dissemination mainly localized in the peritoneal surface has already occurred. The mouse monoclonal antibody MX35 emerged as a vector of interest due to its recognition of the sodium-dependent phosphate transport protein 2b (NaPi2b), overexpressed in this type of cancer. As a first step, the radioimmunoconjugate ^211^At-MX35 demonstrated an interesting therapeutic effect after intraperitoneal administration for the treatment of microtumors of human ovarian cancer cell line NIH:OVCAR-3 [150]. In the perspective of a phase I trial, MX35 F(ab’)_2_ fragment was favored especially because of a higher diffusion into tumors in comparison with the whole Ab and a decrease in immunogenicity risk after injection to patients. Further preclinical investigations clearly confirmed a positive effect on tumor evolution after intraperitoneal injection of doses between 100 and 400 kBq of ^211^At-MX35 F(ab’)_2_ [151,152]. As an alternative treatment protocol to minimize potential systemic toxicity and optimize therapeutic effect, fractionated administration was then tested. A week interval between injections led to an increased efficacy, especially because it allows the recovery of bone marrow [153,154].

^211^At-MX35 F(ab’)_2_ has been used in a phase I study reported in 2009 for the treatment of micrometastases in recurrent ovarian carcinoma. After checking the absence of macroscopic tumor, nine patients were infused by peritoneal catheter with 22 to 101 MBq.L^−1^ in dialysis solution. Even if the results on the pharmacokinetics indicate that therapeutic dose can be delivered to the targeted metastases without signs of toxicity, no real conclusion on therapeutic efficacy can be drawn [155]. An extension of this phase I was continued with the inclusion of three more patients. In this study, estimation of absorbed dose was expanded to all organs and calculations of effective dose were reported. Besides, a new method for the preparation of ^211^At-MX35 F(ab’)_2_ (preconjugation of Ab fragment) allowed to increase the specific activity and administered dose (up to 215 MBq.L^−1^). Overall, the tendency of absorbed dose in organs and the possibility to reach effective dose are consistent with the initial work [156]. Final results after a long-term follow-up of patients for this extended Phase I were recently published. Dose escalation up to 215 MBq.L^−1^ were administered without any sign of radiation induced toxicity meaning that the maximum tolerated dose was not reached. However, regarding effective dose delivered, some doubts were emitted about the diffusion ability of the targeting vector. In these conditions, the risk of recurrence appeared to be non-negligible and instead increasing injected dose, an adjuvant setting was considered [157].

Leukemias (acute myeloid or acute lymphoblastic forms) are hematological malignancies that are difficult to control and existing treatments such as chemotherapy or hematopoietic cell transplantation induce only partial response. As possible alternative, ^211^At-BC8-B10 was developed for targeting CD45, a tyrosine phosphatase protein expressed at the surface of leukemia blast cells. Recently, a cGMP procedure and quality controls were developed for the preparation of astatinated anti-CD45 MAb in the perspective of clinical trials [158]. Recruitment of patients is in progress for Phase I/II studies to determine potential side effects as well as the most efficient dose (NCT03128034). Another investigation of ^211^At-BC8-B10 for the treatment of non-malignant diseases should start the recruiting phase soon (NCT04083183). Recently, two phase I trials were reported for the treatment of another hematologic malignancy, multiple myeloma. Investigations are based on OKT10, a mouse monoclonal antibody with recognizing properties for CD38, a glycoprotein expressed by malignant plasma cells. The radioimmunoconjugate ^211^At-OKT10-B10 previously studied in preclinical phase [130], will also move to clinical studies and will be evaluated in combination with chemotherapeutic agents such as melphalan (NCT04466475) or cyclophosphamide/fludarabine (NCT04579523).

## 4. Bismuth-213

Bismuth belongs to group 15 of periodic classification and exhibits 35 isotopes, most of them having short half-lives (from ns to a few minutes) and only one is considered stable, ^209^Bi, due to a very long half-life of 1.9 × 10^19^ years. Among all these isotopes, only ^212^Bi and ^213^Bi have demonstrated promising features for investigation in targeted alpha therapy.

^213^Bi, a daughter isotope of ^225^Ac, decays by α (2.2%) and β^−^ (97.8%) emission with a half-life of 46 min. It decays to the stable ^209^Bi via successive decays resulting in β^−^ emissions (0.6–2 MeV) and the emission of one α particle (5.9–8.4 MeV). The total emitted energy of ^213^Bi can be attributed at 90% to α-particles and can explain its more pronounced cytotoxicity. In addition, the emission of gamma rays with an energy of 440 keV offers the possibility of imaging by SPECT (Figure 1).

### 4.1. Production

Since ^225^Ac is the parent nuclide, production routes discussed in actinium section are also involved for obtaining ^213^Bi. Indeed, after production, ^225^Ac can be directly used for radiochemistry or adsorbed on a resin to build ^225^Ac/^213^Bi generators. In this case, the coproduction of ^227^Ac with ^225^Ac is not a problem and more production routes may be used. Several types of these generators were developed based on selective separation of ^213^Bi either using cation and anion exchange or extraction chromatography, these options were reported in a detailed review [159]. Nevertheless, the preferred system in clinical studies is based on AG MP-50 cation exchange resin. This generator was first developed by loading a cationic resin with a ^225^Ac chloride solution in dilute hydrochloric acid (1.5 M HCl), but under those conditions, the activity was confined to a small layer at the top of the column and induced radiolytic damage leading to elution problems. Pre-loading of ^225^Ac on a small amount of resin before deposit into the column decreased radiation dose induced to the resin and constant generator production [160]. The process was then slightly improved with the use of columns connected in series that allowed to work with higher activities (2.6 GBq) and minimize ^225^Ac parent contamination. However, this manual method implies prolonged handling steps that appear as the main limitation of this new version [161]. Finally, direct loading of ^225^Ac in 4M HNO_3_ emerged as the best option with a distribution in a larger layer of resin, using automated pump and leading to high elution yields. Due to strong affinity of Bi for halogens and in particular iodide (formation of stable anionic species BiI_4_^−^/BiI_5_^2−^), ^213^Bi can be separated from ^225^Ac adsorbed on AG MP-50 resin using a 0.1 M HCl/0.1M NaI solution as eluent. This method appeared as the most efficient to get ^213^Bi with high purity and in a medium adapted for radiolabeling chemistry. ^225^Ac/^213^Bi generators are often loaded with initial activity between 2 to 4 GBq and the long half-life of ^225^Ac makes possible its use over several weeks with possible elution every 3 h [159]. In order to improve this system and still with the idea to minimize radiolytic damage to the resin, separation of ^213^Bi and ^225^Ac was recently evaluated with two inorganic Isolute resins (SCX and SCX-2). These resins are based on a silica structure grafted with benzenesulfonic acid (SCX) or propylsulfonic acid (SCX-2) moiety allowing extraction of basic compounds from aqueous solution by a cation exchange retention mechanism. Even if an efficient separation could be noticed with lower proportion of residual ^225^Ac after elution, this method would necessitate to use 20% more ^225^Ac than the needed activity as well as a higher amount of resin due to radiolysis leading to a down migration of radionuclides in the column over time [162].

### 4.2. Radiolabeling Chemistry

Unlike some other α-emitters, the existence of a stable isotope facilitates the understanding of bismuth chemistry. The electronic configuration of [Xe] 4f^14^ 5d^10^ 6s^2^ 6p^3^ makes the (+III) oxidation state the most common form of Bi ion, even if (+V) species were also described in specific cases. As a hard Lewis acid, strong affinity for hard donors atoms such as oxygen or nitrogen is expected, meaning that chelating agents such as aminopolycarboxylate ligands would form stable complexes with Bi(III). First chelation work with Bi(III) has been performed with the acyclic chelator DTPA. The fast complexation kinetics as well as the high stability of the corresponding Bi(III)DTPA complex resulted in the development of C-functionalized DTPA derivatives in order to allow potential conjugation to proteins. However, in vivo stability of these precursors was not as high as expected, hampering further preclinical work. On the other hand, the *trans*-cyclohexyl DTPA (CHX-A″-DTPA) ligand demonstrated a very high stability attributed to the pre-organization and rigidity provided by the cyclohexyl moiety. CHX-A″-DTPA ligand emerged as the most adapted to bismuth chelation and plays a key role in the development of ^213^Bi [163]. The macrocyclic DOTA chelator is also appropriate for ^213^Bi chelation, with the formation of highly stable complexes and high kinetic inertness, but the low complexation kinetic appeared initially as a limitation for its use. Indeed, due to the short half-life of ^213^Bi, the reaction time is an important parameter to consider in the radiolabeling chemistry. It was not a problem with DTPA, because of the fast complexation kinetic (5 min at room temperature). But in the case of DOTA complexation, high temperature is often required as well as longer reaction time (30 min). The interest for DOTA was revived with the development of a radiolabeling process using micro waves and allowing complexation of ^213^Bi in only 5 min at 95 °C pH = 9 [20]. Recently, cyclen-based chelators bearing phosphonic or phosphinic arms were described to form ^213^Bi-complexes in suitable reaction conditions (5 min, 25 °C or 95 °C, pH = 5.5), high RCYs, with promising stability in competition with DTPA or in human plasma, but especially at lower ligand concentration than DOTA or DTPA derivatives [164]. Alternately, azacrown ether chelators with pyridine moieties showed similar results with fast complexation under mild conditions, and even if high transchelation in BSA after 3 h (around 50%) can be noticed, no apparent sign of Bi release was measured in a biodistribution study [165].

### 4.3. Preclinical Studies

**Figure 5:**
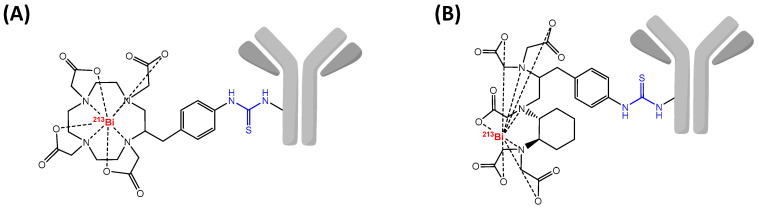
Schematic representation of ^213^Bi-labeled radioimmunoconjugates based on *p*-SCN-Bn-DOTA (**A**) or *p*-SCN-Bn-CHX-A″-DTPA (**B**).

Due to the short half-life of ^213^Bi, direct labeling of pre-modified proteins is favored in comparison with indirect labeling through the use of a radiolabeled prosthetic group. Therefore, among DTPA or DOTA derivatives, C-functionalized versions *p*-SCN-Bn-CHX-A″-DTPA or *p*-SCN-Bn-DOTA are currently the most used for preparation of ^213^Bi-radioimmunoconjugates (Figure 5). Besides, the high temperature required for the labeling of DOTA is not suitable for some sensitive proteins but can be appropriate for heat-resistant vectors such as peptides or small molecules. All of these conditions make that preparation of ^213^Bi-RIC has to be efficient and require a fast tumor targeting. Nonetheless, some strategies are preferred to facilitate this last aspect such as locoregional administration [166], pretargeting [167] or the use of peptides [168].

^213^Bi was one of the first α-emitters to be studied and the initial in vitro investigations in the beginning of the 90s have highlighted the potential of α-particles toward malignant cells. Further work in preclinical models confirmed in vivo stability of ^213^Bi-RIC highlighted by a higher amount of activity measured in the blood pool and no specific uptake in kidneys as expected for free bismuth [169]. Thereafter, a wide range ^213^Bi-RIC was developed with most often mAb as targeting agent [170,171], even if antibody fragments [172] or peptides [173] were also used. Besides, given that ^225^Ac and ^213^Bi have common physico-chemical properties, many radioimmunoconjugates were first developed with ^213^Bi before transposition to ^225^Ac. Finally, several preclinical studies were reported and the most relevant published in the past 5 years are summarized in Table 3.

### 4.4. Clinical Evaluation

Historically, ^213^Bi was the first α-emitter to reach clinical phase with the preparation of ^213^Bi-lintuzumab for treatment of AML. In this Phase I trial, 18 patients with relapsed or refractory AML were treated with 10.36 to 37 MBq/kg of ^213^Bi-lintuzumab. A rapid uptake was noticed in bone marrow, liver and spleen, which are privileged sites of leukemic cells. Absorbed dose ratios between these areas and whole body was measured to be 1000 times more important than analogue radioimmunoconjugates with β-emitters. Even if no complete remission was detected, a significant reduction of marrow blasts was noticed in 14 patients [174]. A phase I/II complementary study demonstrated that sequential administration of cytarabine before treatment with ^213^Bi-lintuzumab injected doses (18.5 to 46.25 MBq/kg) could induce complete remission in some patients. These results are attributed to the cytarabine that reduces tumor volume improving the impact of radiations of ^213^Bi-lintuzumab [175]. Thereafter, ^213^Bi-radioimmunoconjugates (^213^Bi-RICs) were also investigated for therapy of malignant melanoma [176] or on locoregional treatment of bladder cancer [177].

Similarly to ^225^Ac, peptide receptor alpha therapy was developed with ^213^Bi in clinical evaluation. Several phase I studies were reported with ^213^Bi-DOTA-SP for treatment of glioblastoma. First evaluated in two patients, local injection (catheter implanted in the tumor) of a single dose of ^213^Bi-DOTA-SP (375 and 825 MBq) was well tolerated and led in one case to the resection of tumor without long-term recurrence. Another investigation confirmed these first results with a high concentration of activity around the injection site. In addition, magnetic resonance (MR) imaging highlighted the fact that radiations induced necrosis and demarcation of the tumor mass allowing surgical resection. The treatment protocol was then adjusted following the tumor grade with one to seven cycles of ^213^Bi-DOTA-SP in 2-month intervals, in a patient cohort with glioma recurrence. In addition to a decrease in tumor volumes, these studies showed a positive effect on the median overall survival. Even if ^213^Bi-DOTA-SP emerged as a new option of treatment of gliomas, the short half-life of ^213^Bi can be a limit in the homogenous distribution in large tumors. That is in that conditions that longer half-life radioisotope such as ^225^Ac can be of interest [178].

^213^Bi-DOTATOC is another compound that has been evaluated in PRRT in patients with NETs refractory to treatment with β-emitters analogue ^90^Y/^177^Lu-DOTATOC. Seven patients were treated with cycles of increasing activities (1 to 4 GBq) every 2 months in association with the administration of a nephroprotective solution to limit radiation damage to kidneys. Imaging based on 440 keV gamma emission of ^213^Bi allowed to follow the biodistribution of the compound and confirmed the tumor binding as reported for DOTATOC radiolabeled with other radionuclides. Finally, positive responses were noticed in all treated patients with only limited toxicity, which allowed to demonstrated the possibility to overcome therapies resistance with TAT [173]. The analogue ^213^Bi-DOTATATE that could be an alternative in the same pathology model was recently reported but only in preclinical phase [179].

Finally, concerning small molecules as targeting vector, PSMA-617 that represent a major part of clinical investigations with ^225^Ac, was also considered with ^213^Bi. A first administration in a patient with metastatic castration-resistant prostate cancer (mCRPC) that was still progressive after standard therapy was reported. After treatment with two cycles of ^213^Bi-PSMA-617 for a cumulative dose of 592 MBq, significant effect on tumor was noticed by imaging but also by a decrease in PSA level [180]. Dosimetry calculations based on imaging of patients with 68Ga-PSMA-617 and extrapolated to ^213^Bi were performed. Although the estimated dose to tumor was sufficient for clinical applications and that absorbed dose by dose-limiting organs was below values measure for ^225^Ac-PSMA-617, the therapeutic efficacy of ^213^Bi-PSMA-617 appeared to be significantly lower than with the ^225^Ac-labeled analogue [181].

**Table 3:** ^213^Bi preclinical studies involving an identified pathology model (non-exhaustive list).

Preclinical Model	Molecular Target	Targeting Vector	Chelating Agent	Investigation	References
Mouse mammary cancer cells 4T1	Human aspartyl (asparagnyl) β-hydroxylase (HAAH)	PAN-622 (human mAb)	DTPA-maleimide	In vitro, biodistribution, imaging ^111^In-DTPA-PAN-622 and therapy study	[182]
Human ovarian cancer cells (OVCAR-3)	Sodium-dependent phosphate transport protein 2b (NaPi2b)	MX35 (murine IgG_1_ mAb)	*p*-SCN-Bn-CHX-A″-DTPA	Therapeutic efficacy, toxicity and dosimetry	[183]
Human endometrial carcinoma cell line AN3CA	Müllerian-inhibiting substance receptor type II (MISRII)	16F12 (murine mAb)	*p*-SCN-Bn-CHX-A″-DTPA	Biodistribution, dosimetry, toxicity, therapy study in comparison with ^177^Lu-16F12	[184]
				In vitro and therapy study in comparison with ^212^Pb-	[185]
Murine B16-F10 melanoma model	Extracellular melanin	8C3 (murine mAb)	*p*-SCN-Bn-CHX-A″-DTPA	Biodistribution, imaging and therapy study	[186]
		Humanized 8C3 (h8C3)	*p*-SCN-Bn-CHX-A″-DTPA	Biodistribution, imaging, dosimetry and therapy study in comparison with ^177^Lu-h8C3	[187]
Cloudman S91 murine melanoma				Therapy study in combination with anti-PD1 mAb	[188]
Human ovarian cancer cell line SKOV-3 and luciferase analogue (SKOV3.IP1)	HER2	2Rs15d (nanobody)	*p*-SCN-Bn-CHX-A″-DTPA	In vitro, biodistribution, imaging, dosimetry and therapy study	[172]
*Blastomyces dermatitidis* (fungal infection)	(1→3)-β-glucan	400-2	*p*-SCN-Bn-CHX-A″-DTPA	Toxicity and therapy study in dogs	[189]
Human melanoma cell lines M113^WT^ and M113^PD−L1+^	PD-L1	Anti-hPD-L1 mAb	*p*-SCN-Bn-CHX-A″-DTPA	Imaging with ^64^Cu, therapeutic study and toxicity	[171]

## 5. Bismuth-212

**Figure 6:**
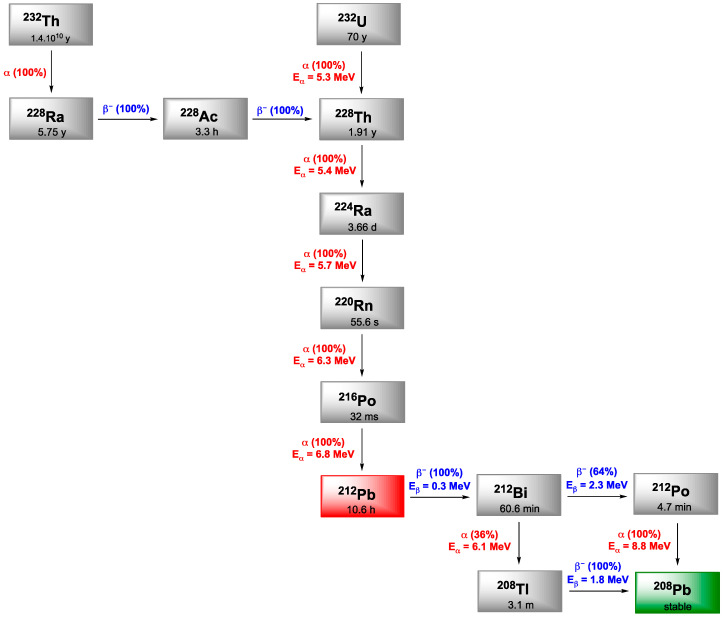
Decay schemes for the production of ^212^Bi and ^212^Pb.

^212^Bi arises from the decay sequence of ^228^Th (1.9 y) and results in ^208^Tl (3.1 min) via 36% of α-emission and by 64% to ^212^Po (0.3 µs) after β^−^-emission, both daughters leading to stable ^208^Pb. ^208^Tl decay occurs through high energy γ-emission (2.6 MeV), which necessitates adapted protection for all the handling phases. Like ^213^Bi, ^212^Bi exhibits a short half-life (60.6 min) which requires rapid targeting as well as efficient production of radiolabeled carrier molecules. On the other hand, in vivo release issues regarding ^212^Bi daughter radionuclides are negligible due to the short decay chain of ^212^Bi to stable ^209^Bi and the short half-lives of the daughters induced (Figure 6).

### 5.1. Production

^212^Bi can be directly recovered from parent radionuclides ^228^Th or ^224^Ra, both being daughters of ^232^Th decay chain. The main production route of ^228^Th is by extraction and purification after natural disintegration of aging ^232^Th [190]. Thereafter, ^228^Th is adsorbed as nitrate complexes on an anion-exchanger column and used as basis for ^224^Ra/^212^Bi generator. In these conditions, separation of the direct daughter ^224^Ra is possible and the eluate is then adsorbed on AG-MP-50, a microporous organic cation-exchange resin, forming the ^212^Bi generator. ^212^Bi can be selectively eluted with diluted HCl (1–0.25 M) or HI (0.05–0.2 M) solutions with low levels of ^212^Pb contamination [191]. In addition to activity levels around 0.7 GBq that can be reached in a production cycle, this method is the only one to be considered as reliable enough for continuous availability of ^212^Bi. Other ^228^Th based generators with deposition on a column of Na_2_TiO_3_ [192] or by incorporation into barium stearate were also developed [193]. Both routes use the short half-life daughter ^220^Rn, either by elution with water into a reservoir, or by vapor condensation into a collection chamber. However, production yields were greatly influenced by radiolytic damages on the purification column or on Ba stearate and did not provide sufficient activity to carry out further radiochemical or preclinical experiments.

### 5.2. Radiolabeling Chemistry

Both bismuth radioisotopes exhibit identical physico-chemical properties, consequently, the radiolabeling chemistry presented previously for ^213^Bi is also valid and the same chelating agents are used. DTPA or DOTA derivatives were often used to form stable complexes with ^212^Bi [163].

### 5.3. Preclinical Studies

^212^Bi was also early investigated in preclinical investigations, especially after validation of stable conjugation of ^212^Bi-DTPA to anti-Tac mAb as well as a high cytotoxicity toward human interleukin 2 (IL-2) receptor positive T-cell leukemia line [194]. This effect of ^212^Bi was also confirmed in several in vitro studies and promoted its interest for specific therapy with α-emitters [195,196]. The tendency was verified after evaluation in murine models of human colon carcinoma [197] or myeloma tumors demonstrated a significant impact on tumor evolution [198]. Later, the possibility to extend ^212^Bi half-life was considered using its direct parent radionuclide ^212^Pb. This approach based on the use of ^212^Pb as an in vivo generator of ^212^Bi clearly demonstrated an effect on tumor evolution without noticeable signs of toxicity [199]. This indirect source of α particles is currently favored instead of the direct use ^212^Bi and may explain why no clinical phase was started.

## 6. Lead-212

Lead is often presented as the heaviest stable element in the periodic classification and exhibits four stable isotopes: ^204^Pb, ^206^Pb, ^207^Pb and ^208^Pb. Among its 38 reported isotopes, five would have appropriate properties for detection (^203^Pb, ^210^Pb, ^211^Pb, ^212^Pb, ^214^Pb) but only two are suitable for clinical application, in imaging with ^203^Pb (SPECT) and therapy with ^212^Pb. ^212^Pb is a β-emitter but its interest in TAT comes from the fact that its first daughter is ^212^Bi (Figure 6). The short half-life of ^212^Bi can be overcome by using ^212^Pb in vivo generator of ^212^Bi.

### 6.1. Production

As parent radionuclide of ^212^Bi, ^212^Pb is also produced following the ^228^Th decay sequence and can be obtained in quite similar ^228^Th or ^224^Ra-based generators. In these systems, the nature of the ion exchange resin and the elution conditions will be the only differences to allow selective separation of ^212^Pb in comparison with ^212^Bi production. In ^228^Th/^212^Pb production route, ^228^Th is adsorbed onto a cation exchange resin (Dowex-50 × 8) in diluted 0.01M HCl. After decay, ^212^Pb and ^212^Bi can be selectively eluted from the column either by 1M HCl (or 0.5M HI) or 0.5M HCl, respectively [200]. Other ^228^Th generators mentioned in the ^212^Bi section could allow the recovery of ^212^Pb, but the problems discussed previously are also a limit for ^212^Pb production. ^224^Ra generators are another option as source of ^212^Pb. As discussed previously, ^212^Bi can be eluted from AG-MP-50 resin with diluted HCl or HI, but ^212^Pb can be eluted using higher acid concentration of 2M HCl. The generator eluate is then evaporated and digested several times with 8M HNO_3_ and finally extracted with diluted 0.1M HNO_3_. After pH adjustment to 5–5.5 with 5M NH_4_OAc, the resulting media can be used for radiolabeling [201]. With this method, between 75 and 90% of the eluted ^212^Pb can be extracted at the end of the process. On the other hand, the generator has to be replaced after one or two weeks due to the short half-life of ^224^Ra.

Alternative strategies based on new supports for ^224^Ra generator allowing an easier separation of ^212^Pb were recently reported. The use of actinide resin (AC or DIPEX^®^ resin) showed the possibility to directly eluate ^224^Ra from a ^228^Th/^224^Ra generator. A supplementary purification step on actinide resin results in the isolation of ^224^Ra in 1M HCl. After evaporation, dissolution in 0.1M HCl and neutralization with 0.5M NH_4_OAc, the corresponding ^224^Ra generator solution in equilibrium with progeny is used for radiolabeling [202]. The use of a Pb-selective extraction resin (constituted of 18-crown-6 ether derivative) is another option for purification of ^212^Pb, washing with 2M HCl is performed to remove other metallic ions and the ^212^Pb^2+^ are directly eluted in acetate buffer pH = 6 [203]. Lastly, ^224^Ra was described to be separated in 0.1M HNO_3_ from parent radionuclides ^228^Th or ^232^U using a bis(2-ethylhexyl) hydrogen phosphate-polytetrafluoroethylene (HDEHP-PTFE) material. After adsorption of ^224^Ra on a cation exchange resin (Dowex 50Wx12), it is possible to selectively eluate ^212^Bi in 0.75M HCl or ^212^Pb in 2M HCl [204].

### 6.2. Radiolabeling Chemistry

**Figure 7:**
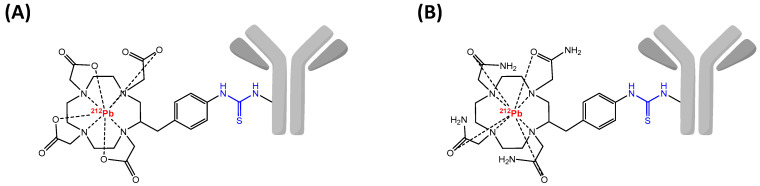
Schematic representation of ^212^Pb-labeled radioimmunoconjugates based on *p*-SCN-Bn-DOTA (**A**) or *p*-SCN-Bn-TCMC (**B**).

Lead belongs to group 14 of the periodic table and is classified as a post-transition metal with a weak metallic character. Its [Xe] 4f^14^ 5d^10^ 6s^2^ 6p^2^ 7s^2^ electronic configuration makes the (+II) oxidation state predominant and to a lesser extend the (+IV) oxidation state. As a borderline acid according to Pearson’s HSAB principle, Pb(II) ion exhibits affinity for donor atoms as nitrogen and oxygen. Even if coordination properties are quite similar to Bi(III), DOTA-based chelators demonstrated a superiority for complexation of Pb(II) in comparison with acyclic chelating agents. The stability of Pb(II)-DOTA was especially validated with in vivo experiments after conjugation to a mAb [205]. However, despite the high stability of the association Pb(II)-DOTA, investigations of the chemical integrity of ^212^Pb-DOTA complex revealed that around 36% of ^212^Bi induced by natural decay of ^212^Pb was released out of the chelator [206]. Besides, in vivo experiments with ^212^Pb-labeled mAb showed that despite a clear tumor accumulation, internalization of ^212^Pb-immunoconjugate under acidic intracellular conditions causes release of ^212^Pb resulting in severe bone marrow toxicity [207]. Thereafter, carboxylate arms of DOTA chelator were substituted by carbamoyl methyl pendent, described to form metal complexes inert to metal release. The resulting TCMC macrocycle (1,4,7,10-tetrakis(carbamoylmethyl)-1,4,7,10-tetraazacyclododecane) was developed to specifically chelate Pb(II) ion, and appeared to be more stable than Pb(II)-DOTA and especially at lower pH. The C-functionalized version *p*-SCN-Bn-TCMC allowed the preparation of TCMC mAb immunoconjugates, and is currently the main structure used for studies with ^212^Pb [208] (Figure 7). Recently, cyclen-based macrocycles with pyridine arms were compared to the two standard of Pb(II) chelation. The structure DOTA-3Py showed similar results than DOTA or *p*-SCN-Bn-TCMC in terms of chelator concentration, RCYs as well as stability in human serum and emerged as a possible alternative for Pb(II) chelation [209].

### 6.3. Preclinical Studies

Despite the stability problems with DOTA discussed above, evaluation of ^212^Pb-103A for the treatment of Rauscher leukemia virus (RVB3) validated tumor targeting with ^212^Pb-RIC, highlighted by a high tumor uptake (58% of the ID/g). Nevertheless, the release of ^212^Pb out of the tumor site and distributed in bones led to a severe bone marrow toxicity that did not allow determination of a therapeutic dose without lethal effects [207]. On the other hand, another investigation for targeting HER2 oncoprotein on ovarian tumors with ^212^Pb-AE1-mAb also resulted in similar toxicity for high doses (0.93–1.48 MBq), but in a complete response in all treated animal after administration of 0.37–0.74 MBq without marked signs of toxicity [199]. However, the therapeutic effect was more marked in small tumors and nearly no inhibition on tumor growth was noticed in larger tumor.

The emergence of TCMC chelating agent led to the development of ^212^Pb-RIC and especially to ^212^Pb-TCMC-trastuzumab that demonstrated promising preclinical results and rapidly reached the clinical phase. A complete strategy from ^212^Pb production to the labeling of proteins was also reported and allowed to facilitate accessibility to ^212^Pb-RIC and clearly contributes to the increasing interest of ^212^Pb in TAT [201]. Based on these principles, two preclinical studies were recently described with ^212^Pb-225.28 for the targeting of chondroitin sulfate proteoglycan 4 (CSPG4) overexpressed in triple-negative breast cancer and ^212^Pb-376.96 to the protein B7-H3 (CD276) in human pancreatic ductal adenocarcinoma (PDAC). Both ^212^Pb-RIC showed promising in vitro results with inhibition of cell lines survival and this tendency was confirmed in in vivo experiments with a high tumor uptake resulting in a positive effect on tumor growth in their respective model [210,211]. Similar results were also described in CD37 positive leukemia and lymphoma models with up to 90% survival of animals after treatment with a single injection of ^212^Pb-NNV003 [212].

Theranostic approaches are based on a unique radiopharmaceutical that can be used for both imaging and therapy. When the pair of radionuclides is different, a difference is often noticed in the biodistribution profile of the RIC, but the use of matched-pair radioisotopes allows to minimize this phenomenon. In that context, ^203^Pb/^212^Pb pair is of interest and is currently being developed with the publication of an increasing number of preclinical studies. First, ^203^Pb and ^212^Pb-radioconjugates were respectively studied for comparison of tissue distribution as potential agent for SPECT imaging and for evaluation of therapeutic efficacy, after association with panitumumab F(ab’)_2_ fragment and Mmelanocortin 1 receptor targeted peptide (MC1L) [213,214]. Thereafter, with the increasing interest for PSMA as promising target for TRT of prostate cancer, PSMA ligands were prepared for chelation of ^203^/^212^Pb. These structures were globally based on the glutamate-urea-lysine sequence known as PSMA inhibitor as well as on functionalized TCMC or DO3AM chelating agents [215,216,217]. The resulting ^212^Pb-NG001, ^203^Pb-CA012 and ^203^Pb-L2 ligands showed a specific tumor uptake in animals with PSMA-positive tumors. Further investigations with ^212^Pb-NG001 and ^212^Pb-L2 demonstrated a positive effect on tumor evolution even if kidneys appeared to be the dose-limiting organ, that point requiring a long-term toxicity evaluation [216,217]. ^203^Pb versions of other PSMA ligands (L1–L5) were tested in small animals for SPECT imaging and allowed tumor visualization consistent with tissue distribution experiments [217]. ^203^Pb-CA012 was also investigated for imaging in biodistribution study, and was administered in two patients with mCRPC for additional dosimetry estimation of therapeutic dose of ^212^Pb-CA012 [215].

### 6.4. Clinical Evaluation

Trastuzumab is a humanized mAb well known to have recognition properties against HER2, a transmembrane tyrosine kinase receptor protein overexpressed in epithelial tumors found in breast, ovarian, pancreatic or colorectal cancers. Already studied with ^213^Bi, the corresponding ^213^Bi-RIC showed encouraging results in a peritoneal model of pancreatic and ovarian cancer. But in some cases, the need to increase the injected dose to get a clear therapeutic effect on animal survival was attributed to the short half-life of ^213^Bi that did not allow a sufficient irradiation time of tumor cells. Consequently, trastuzumab was radiolabeled with ^212^Pb as in vivo generator of ^212^Bi to overcome the difference of half-life between the protein and the radionuclide. ^212^Pb-TCMC-trastuzumab clearly resulted in a significant improvement in survival of animals bearing LS-174T xenografts after intraperitoneal administration of 0.37 MBq (19 vs. 56 days). Median survival was even more improved after multiple injections (up to 3 × 0.37 MBq) at one-month interval (110 days) [218]. Combination with chemotherapy agent as gemcitabine [219], paclitaxel [220] or carboplatin [221] also demonstrated a substantial increase in median survival. These promising data rapidly led to the first phase I study with ^212^Pb-TCMC-trastuzumab in which five dose levels were administered intraperitoneally to patients with primary or recurrent ovarian cancer. Pharmacokinetics data revealed that most of the injected activity remains in the peritoneal cavity, only low redistribution was measured in blood stream and none in other organs. Efficacy of this treatment protocol was highlighted by a reduction of tumor mass as well as the stabilization of tumor progression. The treatment was globally well tolerated, only little toxicity was noticed with some adverse event and no signs of myelosuppression or immune response were detected [222]. Further work on long-term monitoring of the treated patients allowed to confirm that no late toxicity could appear. Besides, a detailed study of several tumor markers identified that TAG-72 serum levels could be a good indicator of tumor response after treatment [223].

^212^Pb-DOTAMTATE (AlphaMedix^TM^) constituted of a TCMC chelating cavity conjugated to the somatostatin analogue octreotate was developed for PRRT of NETs. Preclinical evaluations showed promising results with high uptake in tumor and a full survival in treated animals with doses of 0.74 MBq. Treatment fractionation as well as association with a chemotherapy agent allowed to improve the therapeutic effect of ^212^Pb-DOTAMTATE [224]. These encouraging data led to the start of a clinical trial in PRRT naïve-patients with somatostatin receptor positive NETs. Thirteen patients were included in this phase I evaluation in which three treatment cycles every 8-weeks demonstrated a positive response highlighted by a decrease in tumor size and a well-tolerated treatment with only moderate side effects, even at the highest doses (NCT03466216) [225].

## 7. Radium-223

Radium is an alkaline earth metal originating from the decay process of ^235^U, ^238^U or ^232^Th. Among its 33 known isotopes, only four occur naturally: ^228^Ra (5.75 y) and ^224^Ra (3.63 d) from ^232^Th, ^226^Ra (1600 y) the most abundant, from ^238^U (stable) and ^223^Ra (11.4 d) from ^235^U (703,800,000 y). ^223^Ra and ^224^Ra are both α-emitters but the decay chain of ^224^Ra includes daughter nuclides such as ^220^Rn (56 s) and ^212^Pb (10.6 h) with non-negligible half-lives that could be problematic because of their potential redistribution in the whole organism. Therefore, ^224^Ra has been much less investigated than ^223^Ra, mainly for treatment of noncancerous bone diseases, encapsulation in particles or as generator of ^212^Pb.

**Figure 8:**
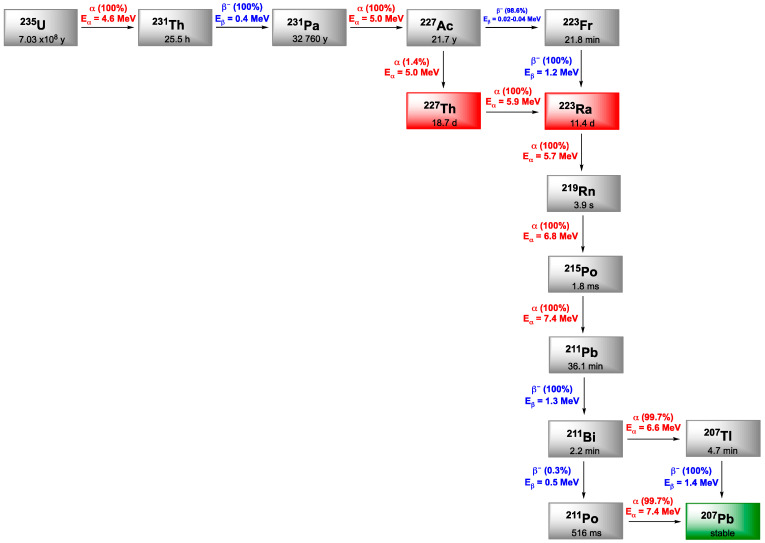
Decay schemes for the production of ^223^Ra and ^227^Th.

^223^Ra decays to ^219^Rn (3.96 s), ^215^Po (1.78 ms), ^211^Pb (36.1 min), ^211^Bi (2.14 min), ^207^Tl (4.77 min) or ^211^Po (0.52 s) and stable ^207^Pb. This long disintegration sequence generates four high energy α particles, two β^-^ particles and γ rays conducing to a total energy emitted around 28 MeV. The cascade of α particles generated (around 96% of the total energy) allows to increase the radiation dose received by the targeted tissues, whereas low energy γ component (269 keV) is of interest for monitoring (Figure 8).

### 7.1. Production

Whereas ^223^Ra can be recovered from decay of ^235^U (or one of its daughter nuclide) through the cascade ^235^U, ^231^Th (25.5 h), ^231^Pa (3.28 × 10^4^ y), ^227^Ac (21.7 y) and ^227^Th (18.7 d), this approach based either on the exploitation of natural uranium ore (composed of 0.7% of ^235^U) or on its daughter ^231^Pa, remains quite limited. Another possibility is from ^227^Ac decay, which substantially improved ^223^Ra availability especially through the development of ^227^Ac-^227^Th-^223^Ra generator. The ^227^Ac necessary for this procedure is mainly produced by thermal neutron irradiation of ^226^Ra targets following the ^226^Ra (n,γ)^227^Ra—^227^Ac reaction (in nuclear reactor) [226,227] or to a lesser extent by proton irradiation of natural ^232^Th targets. For this latter method, a 10-day irradiation using an intense proton beam of 800 MeV demonstrated the possibility to generate ^227^Ac through the ^232^Th (p,x)^227^Ac reaction. In these irradiation conditions, it has to be mentioned that direct production of ^223^Ra (^232^Th (p,x)^223^Ra reaction) and production of ^227^Th (^232^Th (p,x)^227^Th reaction) are also possible [22]. Similar work was also reported from proton irradiation with a lower energy (90–135 MeV) and confirmed the interest in ^227^Th production whereas direct production of ^223^Ra in those conditions was not significant [228]. However, despite these few production routes, accessibility of ^227^Ac and ^227^Th is still limited and others alternatives based on recycling of actinium/beryllium neutron source [229] or by treatment of production waste generated after cyclotron production of ^225^Ac [230] were also reported.

Beyond production route of ^223^Ra, separation of the ^227^Ac-^227^Th-^223^Ra mixture is also an important step in order to recover ^223^Ra with an appropriate purity for biomedical applications. The difference in electrical charge between ^227^Ac^3+^, ^227^Th^4+^ and ^223^Ra^2+^ cations results in the formation of negatively charged species with nitrate ions that facilitate their separation by using ion exchanger materials. In the first reported method, a mixture of ^227^Ac and ^227^Th in 1M HCl isolated from a ^231^Pa solution was deposited on a column packed with a strong anion-exchange resin based on P,P’-di(2-ethylhexyl)methanediphosphonic acid moiety (Dipex-2). While ^227^Ac and ^227^Th are selectively retained, ^223^Ra can be recovered with 1M HNO_3_ and concentrated on an AG 50W-X12 cation exchange resin to be recovered in 8M HNO_3_, evaporated to dryness and dissolved in a sodium chloride/sodium citrate solution before use [231]. Other purification processes by anion exchange in a methanol/water nitric acid solution were also developed, using an AG1-X8 column allowing the selective separation of ^223^Ra (with a 4:1 MeOH/1M HNO_3_ mixture), ^227^Ac (with 8M HNO_3_) or ^227^Th (with 0.5M HCl) [229], or a Dowex 1 × 8 resin for recovering ^223^Ra (with a 4:1 MeOH/2M HNO_3_ mixture) [230]. An alternative method was reported which consisted in the isolation of ^223^Ra after elution on a Dowex 1 × 8 resin with a 4:1 MeOH/0.7M HNO_3_ mixture, then using a strong cation exchange column with Dowex 50 × 8 in order to carry out ^223^Ra in an EDTA-saline solution (Na_2_EDTA-NaCl at pH = 7.4–8) allowing a direct administration after purification [232].

It has to be mentioned that ^227^Th is also obtained from ^227^Ac decay and that similar methodology is often used for separation from residual ^227^Ac or daughters such as ^223^Ra. Usually, the corresponding mixture in solution in 7M HNO_3_ is deposited on an AG1 × 8 anion exchange chromatography resin, and ^227^Th is selectively retained when other radionuclide have eluted from the column. ^227^Th is then recovered in 12M HCl, evaporated to dryness before dissolution in 0.1M HNO_3_ to be used for radiochemistry [233]. The ability to purify ^227^Th from daughters or residual ^227^Ac was also demonstrated by other unusual methods such as extraction chromatography columns (TEVA or UTEVA resins) [234] or micro-spin column with cation exchange resin for a direct elution in buffers compatible with antibody radiolabeling [235,236] (see the Thorium section).

### 7.2. Radiolabeling Chemistry

Radium belongs to group 2 of the periodic table and consequently exhibits similar chemical properties to its lighter homologues magnesium, calcium and barium. With a [Rn] 7s^2^ electronic configuration, the corresponding divalent cation Ra^2+^ is the only species formed. Concerning its potential chelation, as a hard acceptor, a more pronounced affinity to hard donor atoms such as oxygen is expected. However, only few chelating agents including DOTA, Kryptofix 2.2.2 or calix [4]-tetraacetic acid have been studied for chelation of ^223^Ra^2+^ [237,238]. Even if the latter demonstrated the ability to form the most stable complex, it remains anecdotal and coordination chemistry of radium is still quite limited. Consequently, the lack of an efficient chelating agent do not allow conjugation to biomolecules as other α-emitters, and even if retention in structures such as nanoparticles is possible, ^223^Ra is mainly used in its chlorine salt form [^223^Ra]RaCl_2_.

### 7.3. Preclinical Studies

Bone tissues are mainly composed of osteoblasts which are cells involved in bone formation by production of the mineral component hydroxyapatite Ca_10_(PO_4_)_6_(OH)_2_, and osteoclasts which are cells involved in bone degradation. In healthy bone tissues, a balance between osteoblasts and osteoclasts allows regular bone regeneration, but in the case of bone metastases, cancer cells disturb this cycle and favor the production of osteoblasts. The stimulation of osteoblasts creates zones of high consumption of substrates necessary for the formation of hydroxyapatite [239,240]. Due to its chemical properties similar to calcium, Ra^2+^ naturally targets this mineral component, being incorporated by substitution with calcium ion in the bone matrix. Under those conditions, a supply of radium would increase its possible inclusion into hydroxyapatite. Consequently, conjugated to its bone-seeking properties, radiations of ^223^Ra appeared as a promising option for therapy of skeletal metastases [241].

The first in vivo investigations confirmed the potential of ^223^Ra with a rapid clearance from blood and soft tissues by intestinal absorption, as well as a significant accumulation and retention on bone surfaces. The evaluation in nude rats grafted with MT-1 cells, a breast cancer cell line known to cause skeletal metastases, was the first to highlight the antitumor effect of ^223^Ra [242]. Significant survival was observed in animals treated with activities ≥10 kBq (corresponding to 100 kBq/kg) without any signs of toxicity (on bone marrow or body weight loss). In comparison with commercial β-emitters bone-seekers such as ^89^SrCl_2_ (Metastron) or ^153^Sm-EDTMP (Quadramet), a higher selective uptake was noted for ^223^Ra^2+^. Even if an accumulation in some soft tissues such as spleen or kidneys was also measured, the high specificity of ^223^Ra could allow to decrease the injected dose in order to minimize this effect. Besides, due to the lower cross-fire effect of α particles, the radiation dose is more localized to bone surface and allows to better preserve bone marrow compared to β-emitters [243]. A complementary work for the evaluation of potential toxicity of ^223^Ra was performed in mice after intravenous administration of high dose levels (1250, 2500 or 3750 kBq/kg). This study pointed out the tolerance of bone tissues to these levels of radiation with only a low dose-related effect on hematopoietic cells [244].

After in vivo administration of ^223^Ra, questions about the outcome of decay products can naturally be raised. The long half-life of ^223^Ra allows a deeper inclusion into bone mineral, in a greater proportion, and an elimination of the fraction that has not been fixed (avoiding irradiation of soft tissues). In these conditions, redistribution of daughter nuclides is minimized because they become trapped in bone tissues. Besides, the short half-life of the first daughter ^219^Rn (3.96 s) is also an element for fewer chances of migration out of the bone. That is for these reasons that ^223^Ra was mainly favored in regards to ^224^Ra that exhibits a half-life of 3.63 days and decays to ^220^Rn with a half-life of 56s. Animals experiments with ^224^Ra showed that a significant fraction of ^220^Rn diffused away from bone site, when redistribution was only estimated below 1% of the total activity in bone for ^223^Ra [245,246]. Nevertheless, ^224^Ra shows interesting therapeutic properties and was one of the first α-emitters to be used in clinical application for treatment of ankylosing spondylitis for a while, but questions about the dose administered as well as doubts about the development of others malignancies in treated patients resulted to the market withdrawal of ^224^Ra dichloride [247,248]. Unlike ^223^Ra, ^224^Ra appears no longer in use under its dichloride form, however, it has been described as generator of ^212^Pb/^212^Bi [202], or in others strategies to prevent redistribution phenomenon described above by use of ^224^Ra in solution with a chelating agent EDTMP for retention of ^212^Pb [249] or by encapsulation in microparticles [250] or liposomes [251].

### 7.4. Clinical Evaluation

In early-stage prostate cancer, tumor cells are only located in the prostate and can be more easily treated by surgery, external beam radiations or brachytherapy. When advanced phases are diagnosed, metastatic dissemination in bone tissues is frequently observed, and others treatment options such as chemotherapy or more often hormone therapies are applied. This latter strategy allows to slow cancer cells growth and limit the spread of metastases by the use of blocking agents of androgenic hormones production. Unfortunately, in most cases, malignant cells develop a resistance to this form of treatment meaning that the pathology evolves to a mCRPC. That is in this specific model of bone metastases in mCRPC that ^223^RaCl_2_ was mainly evaluated.

The promising results shown by ^223^Ra in the preclinical phase resulted rapidly in a phase I study in 25 patients with bone metastases from breast and prostate cancer. Single injections of several dosage levels (46, 93, 163, 213 and 250 kBq/kg) were administered to patients and showed a clear effect on bone metastases evolution, especially on the reduction of alkaline phosphatase, a marker of bone cancer development. As conclusion, a median survival of patients was evaluated to be over 20 months and a phase II study was considered. Only reversible myelosuppression and mild to moderate side effects including diarrhea, bone pain or fatigue were noticed [252]. More specific studies on pharmacokinetic and biodistribution aspects confirmed the first tendency observed with a rapid blood clearance, low hematologic toxicity and a clear effect on several biomarkers of tumor evolution such as PSA, alkaline phosphatase and serum N-telopeptides [253]. A phase II randomized study evaluated the combination of external-beam radiotherapy with multiple injections of ^223^Ra (4 × 50 kBq/kg) every four weeks in 64 patients with mCRPC. Like the phase I conclusions, a significant effect was also noticed on blood biomarkers (decrease in alkaline phosphatase and PSA concentrations). This treatment option also allowed the prevention of skeletal-related events (complication due to bone metastases such as bone pain, risk of death…) still without detection of signs of toxicity. From these results, the possibility to increase the injected dose and to extend the treatment duration were mentioned in order to improve the response [254]. After that, the Phase III ALpharadin in SYMptomatic Prostate CAncer patients (ALSYMPCA) study with a large cohort of patients with mCRPC (n = 921) was started to definitively validate the promising results from the previous phases. In this work, an improvement of overall survival of 3.8 months was determined with a decrease of 30% in risk of death in comparison with placebo group. Finally, it confirmed the interest in ^223^Ra treatment and also opened new prospects for a possible association with chemotherapy agent [255,256].

Following these clinical evaluation phases, ^223^RaCl_2_ (Xofigo^®^; Bayer AG, Berlin, Germany) was validated by FDA in 2013 for treatment of bone metastases in cases of mCRPC, and became the first radiopharmaceutical approved for TAT. As mentioned above, due to its different mechanism of action in comparison with chemotherapy agents, the possibility of an association could offer a better treatment efficiency. Several combinations are currently studied in phase I or phase II trials, especially with enzalutamide (an androgen receptor signaling inhibitor, NCT03305224), pembrolizumab (a monoclonal antibody against PD1 protein, NCT03093428), niraparib or olaparib (both are inhibitors of poly-ADP-ribose polymerase, NCT03076203 or NCT03317392). Even if advanced prostate cancer is the main pathology targeted with ^223^Ra, this radionuclide is under investigation in others pathologies associated with bone metastases such as breast or renal cancer [257,258]. The work on clinical applications with ^223^Ra is considerable and detailed reviews can provide more information about the global state of art on ^223^Ra but also on the ongoing clinical trials [259,260,261].

## 8. Terbium-149

**Figure 9:**
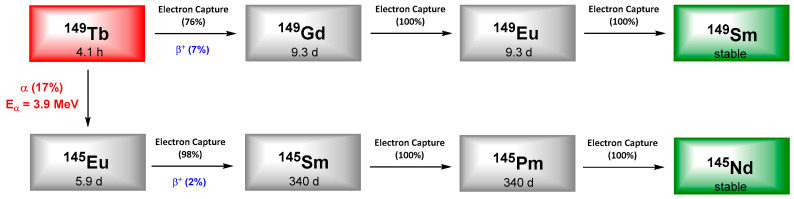
Decay scheme of ^149^Tb.

Terbium is a rare-earth metal with only one stable isotope, ^159^Tb, whose physico-chemical properties are interesting for the development of fluorescent complexes. This element is also described as the “Swiss army knife of nuclear medicine” because among its thirty-six isotopes reported, four of them exhibit suitable properties to cover all modalities in nuclear medicine: ^152^Tb (17.5h, E_β+_ = 1.08 MeV) for positron emission tomography (PET) [262,263], ^155^Tb (5.32d, E_γ_ = 86.55 keV) for SPECT and Auger electron therapy [264], ^149^Tb (4.1h, E_α_ = 3.97 MeV) and ^161^Tb(6.89 d, E_β-_ = 0.154 MeV) for therapy [265,266]. ^149^Tb decays to several radiolanthanides by emission of low energy α (3.97 MeV, 17%), electron capture (76%), and β^+^ particles emission (730 keV, 7%), making it interesting for TAT and a possible follow-up by PET. It has to be mentioned that the potential radiotoxicity of the daughter isotopes (long half-life) generated is still to be determined (Figure 9).

### 8.1. Production

Three main production routes have been reported: (i) via the ^152^Gd(p,4n)^149^Tb reaction [267,268,269]. This light particle induced reaction on ^152^Gd requires a proton energy beam of around 50 MeV and can be performed in commercial high energy cyclotrons (70 MeV). In addition to high production yields able to supply a possible clinical use, the conditions of production are quite easily available, making this route very promising. However, the main drawback of this method is that natural Gd is not adapted for the production and the material used has to be highly enriched for a more efficient production process [270]; (ii) via the ^181^Ta(p,spall)^149^Tb reaction followed by mass separation. ^149^Tb is often produced using spallation reaction of high-energy protons (1.4 GeV) to a tantalum target. This method is also very reliable and could allow to produce large quantities of ^149^Tb (Ci level) [267]. The main drawback is the cost of high-energy accelerator and the current limited efficacy of mass separation; (iii) via heavy ion induced nuclear reactions. Among this category, the indirect route consists in the irradiation of a neodymium target by a ^12^C-ion beam generating ^149^Dy (4.20 min) that decay to ^149^Tb (^142^Nd(^12^C,5n) ^149^Tb reaction) and the direct route is the ^141^Pr(^12^C,4n)^149^Tb reaction. Even if the indirect route showed more interesting yields than the direct one, production yields and radiochemical purity reported were very low in comparison with the two first routes mentioned. Besides, the availability of accelerators equipped with a ^12^C-ion beam is quite limited [267,271,272].

Recently, another production route based on the irradiation of an ^151^Eu target with a ^3^He-ion beam via the ^151^Eu(^3^He,5n)^149^Tb reaction was reported. The advantages are a wider availability of the target material (^151^Eu is more abundant than ^152^Gd) and a much easier radiochemical process with the possibility to remove ^151^Eu after irradiation in aqueous solution as a ^151^Eu(II) species. However, the main limitation is the ability to have access to an equipment with a high intensity ^3^He-beam [273,274,275].

Whatever the production route, reactions are nonspecific and induce contaminations by others terbium isotopes (such as ^150^Tb and ^151^Tb), daughter isotopes (^145^Eu or ^149^Gd) or byproducts (^133^Ce or ^133^La), that cannot be removed by conventional purification methods. The isotope separation process (high precision mass spectrometry, ISOLDE facility at CERN) is currently used to get ^149^Tb with high purity. During or after irradiation, radiolanthanides spread out of the target by heating and are ionized before separation according to their mass-to-charge ratio using online mass separator or off-line system respectively. The radionuclide of interest are recovered on aluminum or zinc coated gold foils (inert support) that are then dissolved in hydrochloric or nitric acid. Then, the resulting solution is adsorbed on a cation exchange resin that allows for separation of isotopes by slow elution with α-hydroxyisobutyric acid. Finally, the radionuclide is obtained in an appropriate media for radiochemistry.

### 8.2. Radiolabeling Chemistry

Generally, lanthanides are found in the (+III) oxidation state in solution. Chemical properties of Ln^3+^ ions are mainly ruled by the 4f valence electrons. Being hard acceptor species, the chelating agent should be comprised of hard coordination sites (oxygen or nitrogen Lewis bases) like carboxylic acid, phosphonates, amine or amine derivatives. Consequently, multidentate acyclic (DTPA derivatives) or macrocyclic (DOTA derivatives) chelators are appropriate for a stable coordination of this radioisotope. DTPA is one of the first acyclic chelator that has been studied in radiochemistry and that has demonstrated adequate chelation properties with many (radio)metals. However, it was overtaken in terms of stability by a second generation of derivatives such as CHX-A″-DTPA (stability improved by the rigidity brought with cyclohexane moiety) even if they are still considered as less stable than complexes from DOTA macrocycle. Therefore, functionalized derivatives *p*-SCN-CHX-A″-DTPA and *p*-SCN-Bn-DOTA were preferred in preclinical studies with ^149^Tb.

### 8.3. Preclinical Studies

It was in the 90s, with the emergence of interest in α-emitters that the real potential of ^149^Tb was detected and its development encouraged [276]. While ^213^Bi was already in clinical trials, and was presented as a reference, a comparative study reported the targeting of a mutated E-cadherin adhesion protein (d9) only expressed in gastric cancers. Both ^149^Tb and ^213^Bi-radioconjugates showed similar immunoreactivity fractions (respectively 30% and 35%), as well as a very low internalization rate. Despite some differences in isotopes properties (half-lives, decay chain, or LET), a clear effect on cells proliferation (MDA MB 435S human breast cancer cells transfected with mutated d9 E-cad) was observed with both immunoconjugates [277].

The same chelating unit was then associated with the monoclonal antibody rituximab (^149^Tb-rituximab) for the targeting of CD20 receptors expressed at the surface of B cells in Non-Hodgkin Lymphoma (NHL) or chronic lymphocytic leukemia. For this first preclinical study, a lethal number of Daudi cells (derived from a human Burkitt lymphoma) were xenografted into severely combined immune-deficient mice. ^149^Tb-rituximab was injected three days after grafting in order to specifically target the circulating single cancer cells. Treatment with a single injection of 5.5 MBq of ^149^Tb-rituximab demonstrated a significant increase in survival (around 89%) over 120 days, with no signs of toxicity in comparison with control groups. Besides, this study also provided information about the in vivo fate of ^149^Tb daughter nuclides. At early time points the majority of the radioactivity was located in organs with high blood pool such as spleen, heart, kidney and also liver. However, after four months, more than 70% of the initial dose was excreted and the resulting radioactivity from daughter nuclei was measured in bone tissue and liver, as expected for free lanthanide. Authors suggested that the injection of chelating agents such as EDTA or DTPA just after treatment could reduce this amount [278].

^149^Tb as well as its three others isotopes of interest ^152^Tb for PET, ^155^Tb for SPECT and ^161^Tb for therapy with low energy α particles, were the object of an interesting investigation in which a new construct (cm09) was developed including a DOTA as chelating moiety, associated to folic acid as vector for targeting folate receptor-positive cancer cells (involved in many types of cancers such as breast, colorectal, renal or ovarian cancer) and to 4-(*p*-iodophenyl) butyric acid, to enhance affinity with human serum albumin for extending the blood half-life of the conjugate [279]. ^161^Tb being more easily available, most of the experiments (stability in human serum, in vitro and in vivo) were done with this isotope and ^149^Tb was used only in the therapy study. An increasing uptake in tumor over 24 h as well as a fast global clearance resulting in high tumor to background ratios were observed in the biodistribution study. The only drawback detected was a quite high and prolonged uptake in kidneys. Therapy study was performed with two injections of ^149^Tb-cm09, 1.1 MBq at day 0 and 1.3 MBq at day 4 or a single injection of ^161^Tb-cm09, with 11 MBq activity calculated to get a quite similar absorbed dose in tumor in comparison with ^149^Tb-cm09. A prolonged survival time was observed in both protocols even if the effect seemed to be slightly clearer with ^161^Tb-cm09. However, some limiting factors of this study (such as availability of ^149^Tb or limited number of animals involved) do not allow to conclude on the real effect of these two radioconjugates. On the other hand, ^152^Tb-cm09 and ^155^Tb-cm09 allowed researchers to get satisfying images of xenografted tumors in mice in PET and SPECT modalities, respectively. Using exactly the same probe and element, this work demonstrated the possibility to perform imaging, but especially therapy offering alternatively α (for single cells) or β^−^ (large tumors) particles or even to consider a cocktail for a better efficacy. Overall, it confirmed the potential of these four terbium isotopes for clinical applications [279]. In order to support these first results, supplementary experiments were published few years later. The radiosynthesis and stability of ^149^Tb-cm09 as well as in vitro effect (activity dependent inhibition) on viability human KB cancer cells via folate receptor specificity, were validated. Concerning the therapy part, a quite different procedure was selected with a single injection of 1.5 or 2.2 MBq of ^149^Tb-cm09 in mice bearing KB tumor model. It resulted in an inhibition of tumor growth of 62% and 85% leading to an increase in the survival time of 45% and 105%, respectively. No particular toxicity was observed in blood plasma parameters even in the kidneys, an organ that showed a non-negligible uptake in the previous study. Despite these encouraging results, more toxicity data about the impact of ^149^Tb-cm09 on kidneys are necessary. Dosimetric calculations showed that absorbed dose by the tumor in the two groups treated with ^149^Tb-cm09 was slightly lower than the calculated dose for 10 MBq of ^161^Tb-cm09 (19 or 26 Gy vs. around 33 Gy) tested in the first series of experiments. Potential absorbed dose and observed effect on tumor growth indicate an improved impact of alpha therapy in comparison with beta. However, it is obvious that this tendency must be confirmed with further investigations involving similar dose of ^149^Tb- and ^161^Tb-radioconjugates [280].

Finally, as with ^225^Ac or ^213^Bi, ^149^Tb was recently investigated for the treatment of metastatic castration-resistant prostate cancer (mCRPC) using the small ligand PSMA-617 [281]. Even if ^225^Ac-PSMA-617 and ^213^Bi-PSMA-617 showed very promising results, ^149^Tb exhibits interesting physical properties as an intermediate half-life and especially, a decay chain without the emission of additional α that could result in irradiation of non-targeted tissues.

Based on ^177^Lu-PSMA-617 biodistribution data, calculations of the distribution in tumor, blood kidneys and liver as well as corresponding ratios resulted in values generally higher than with ^213^Bi-PSMA-617, but quite lower than ^225^Ac-PSMA-617. Estimations of the absorbed dose of ^149^Tb-PSMA-617 to tumor appeared to be higher than that of ^177^Lu-PSMA-617. This tendency was similar with the dose to kidney estimated about 10 times higher than with ^177^Lu-PSMA-617. Nevertheless, this dose remains below the well-tolerated limit of 23 Gy reported in previous therapy study with ^177^Lu and could correspond to an equivalent of six injections of 6 MBq of ^149^Tb-PSMA-617. The therapeutic study was led on mice xenografted with transduced PC-3 human prostate cancer cells (PC-3 PIP) to express high levels of PSMA. The procedure was performed with four groups: a control group, a group receiving a single injection of 6 MBq of ^149^Tb-PSMA-617, a group with 2 × 3 MBq of ^149^Tb-PSMA-617 at day 0 and day 1, and the last group with 2 × 3 MBq of ^149^Tb-PSMA-617 at day 0 and day 3. In comparison with control group, a significant delay in tumor growth as well as an increased median survival were observed for treated mice, even if a better effect was noticed for groups that received a fractionated dose. No sign of toxicity was measured in blood plasma parameters, body weight or organ mass in all tested groups.

β^+^ particles emission of ^149^Tb makes the ^149^Tb-conjugates particularly interesting for imaging. The proof-of-concept of this “alpha-PET” approach was demonstrated with ^149^Tb-DOTANOC in mice xenografted with pancreatic cell lines and resulted in high quality PET images [282]. It has been further developed in this study with the injection of 5 MBq of ^149^Tb-PSMA-617 in mice with PC-3 PIP tumors. A selective accumulation was noticed in tumors and concerning normal tissues, high uptake in bladder (due to the renal excretion) and to lesser extend in kidneys. The possibility of the use of ^149^Tb-radio(immune)conjugates in PET imaging is a real advantage over others α emitters [281].

Overall, ^149^Tb demonstrated an obvious potential for TAT. However, its availability is the main limitation to its wide development especially because the technology necessary for its production (high energy accelerators and mass separation system) is specific and still needs improvements to reach the potential needs. This partly explains why only few preclinical studies were reported and to our knowledge, no clinical trials have started yet.

## 9. Thorium-227

Thorium is a metal of the actinide series whose isotopes are all radioactive. Natural thorium exists almost exclusively as ^232^Th and to a lesser extend ^230^Th. Thorium isotopes exhibit mostly very short (few μs or ms) or long (few years) half-lives and only two exhibit a potential for TAT: ^226^Th (30.6 min) and ^227^Th (18.7 days). Nevertheless, due to a difficult production process (from ^230^U decay), availability of ^226^Th remains presently anecdotal with respect to the amount needed for research, and ^227^Th emerged as the most promising candidate.

### 9.1. Production

As reported in previous section, ^227^Th is the precursor of ^223^Ra through the emission of a 5.9 MeV α-particle. Therefore, decay characteristics, properties as well as production routes detailed for ^223^Ra are also valid for ^227^Th.

### 9.2. Radiolabeling Chemistry

The electron configuration of Th is [Rn]6d^2^7s^2^. Consequently, Th(IV) is the only stable oxidation state in aqueous medium. Similarly to ^223^Ra, free ^227^Th exhibits appropriate characteristics for incorporation into hydroxyapatite, resulting in a natural affinity for bones. Unfortunately, its use as a bone targeting agent is not appropriate because of uptake in soft tissues such as kidneys (renal clearance), liver or spleen. On the other hand, unlike ^223^Ra, ^227^Th can form stable complexes with appropriate chelating agents. In order to better manage thorium radiations, its association with phosphonate derivatives that are well known to have similar targeting abilities to bone tissue were investigated first. ^227^Th-complexes with polyphosphonate ligands such as diethylene triamine *N,N′,N″*-penta(methylene)phosphonic acid (DTMP), 1,4,7,10-tetraazacyclododecane *N,N′,N″,N‴*-1,4,7,10-tetra(methylene)phosphonic acid (DOTMP) and ethylenediamine-tetramethylenephosphonic acid (EDTMP) demonstrated a high and selective bone uptake, quite long retention in bones as well as promising in vivo stability [233,283].

Like other radionuclides, Th(IV) is stably chelated by DOTA, allowing for conjugation to a biomolecule from a functionalized DOTA. This method was largely applied to ^227^Th using *p*-isothiocyanatobenzyl-DOTA. Early studies reported the preparation of ^227^Th-DOTA complex at pH = 5.5 and 55 °C for 40 min followed by the conjugation step with two different antibodies (rituximab and trastuzumab). This two-step procedure showed overall radiochemical yields from 6% to 17% with at least 1 mg of antibody [284]. This procedure reflects issues usually encountered with DOTA-based chelating agents, namely use of an elevated temperature incompatible with heat-sensitive proteins and which implies a two-step procedure. Consequently, it was necessary to make the radiolabeling procedure more efficient and particularly workable at milder reaction conditions (i.e., room temperature).

Inspired from siderophores (natural chelators with high affinity for iron), many synthetic analogs were developed for the chelation of actinides ions for applications as in vivo decorporation agents. The hydroxypyridinone moiety (HOPO) emerged as one of the most efficient chelating unit for binding actinides. More precisely, the 1,2-HOPO or 3,2-HOPO isomers are of interest and allow to get a promising range of chelators after grafting on a polyamine scaffold. Several polydentate HOPO ligands demonstrated their efficacy and selectivity for chelation of some lanthanides and actinides as well as low in vivo toxicities [285,286,287]. Thorium complexes confirmed interesting chelation properties with high stability constant [288,289]. This preliminary work resulted in the development of the octadentate ligand 3-hydroxy-*N*-methyl-2-pyridinone (Me-3,2-HOPO) bearing a carboxylic arm for conjugation to biomolecules. In addition to a faster and more efficient ^227^Th complexation than DOTA, complex showed fast and complete blood elimination after 24h, excretion through the hepatobiliary system, and more importantly, no significant radiation dose to bones, sign of a good in vivo stability [290,291]. This new chelating agent makes possible the direct labeling of modified antibodies after conjugation to ε-amino groups of lysine residues with usually around one ligand grafted per antibody [292]. Grafting of chelating unit as well as radiolabeling did not impact the recognition properties of the antibody and the resulting ^227^Th-immunoconjugate demonstrated a high in vitro stability over at least 48h. It has to be mentioned that from 2016 the use of this chelator was favored for preclinical and clinical studies.

A comparative study of chelating agents containing HOPO or picolinic acid (pa) moieties was recently reported for possible complexation of ^226^Th. Chelation properties of octadentate HOPO ligands were confirmed and octadentate or undecadentate pa ligands appeared as potential chelators for thorium by demonstrating high radiolabeling yields and interesting stability of the corresponding complexes [293].

### 9.3. Preclinical Studies

First preclinical evaluations were performed with ^227^Th-*p*-benzyl-DOTA-rituximab (or ^227^Th-rituximab) for the targeting of CD20 receptors expressed at the surface of B cells in Non-Hodgkin lymphoma (NHL) or leukemia. Further investigations validated properties of ^227^Th-rituximab towards several CD20 positive lymphoma cell lines (Raji, Rael and Daudi cells) [284,294,295]. Association with trastuzumab (Herceptin^®^) for the targeting of human epidermal growth factor receptor-2 (HER2/neu), a tyrosine kinase receptor that is overexpressed in more malignant forms of metastatic breast or ovarian cancers, represents another consequent part of ^227^Th investigations. Other reports include ^227^Th-*p*-benzyl-DOTA-trastuzumab (or ^227^Th-trastuzumab) on HER2-expressing cell lines from breast and ovarian cancer models [296,297,298].

La protein is involved in important biological processes such as transcription and translation. After DNA damage induced by radiations or drugs, this protein is highly expressed by cells in late stages of apoptosis and appeared as a promising target for necrotic tumor cells. Conjugated to the La-specific murine mAb DAB4 (APOMAB^®^), administration of ^227^Th-DOTA-DAB4 as monotherapy or in combination with chemotherapy (gemcitabine and cisplatin) was studied in mice bearing Lewis Lung 2 tumors. While monotherapy showed a moderate antitumor activity, association of treatments resulted in higher responses whatever the injected dose of ^227^Th-DAB4 (5, 10 or 20 kBq). These interesting results being explained by a “cascade reaction” with an effect of chemotherapy leading to cells in apoptosis process, easier to target with ^227^Th-DAB4 [299].

**Figure 10:**
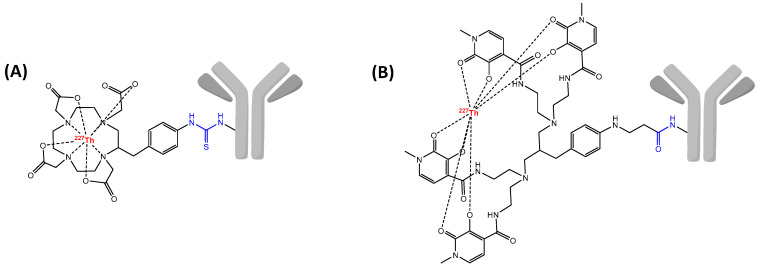
Schematic representation of ^227^Th-labeled radioimmunoconjugates based on *p*-SCN-Bn-DOTA (**A**) or Me-3,2-HOPO (**B**).

Thereafter, a new generation of ^227^Th-RIC emerged with the development of a more efficient chelating unit bearing 3,2-HOPO moieties. Associated to the humanized mAb lintuzumab, the corresponding targeted ^227^Th conjugate (^227^Th-lintuzumab or CD33-TTC) was studied for the targeting of CD33, a sialic acid transmembrane receptor expressed in blood cancers and especially acute myeloid leukemia. Therapeutic efficacy of ^227^Th-lintuzumab (or CD33-TTC) in subcutaneous or disseminated HL-60 model was first investigated [292]. Among the wide range of biomarkers targeted, CD70, a transmembrane glycoprotein from the tumor necrosis factor (TNF) family naturally expressed in T-cells, deserved to be mentioned. Its receptor-ligand interaction with CD27 is known to play a key role in immune response. Overexpression of CD70 receptor was associated to the development of lymphomas or even solid cancers such as renal cell carcinoma. Dose escalation in 786-O xenografted mice showed promising results with a significant antitumor activity illustrated by an increase in median survival and with minimum 60% of animals still alive in all groups after the study time (131 days) [300]. Finally, fibroblast growth factor receptor 2 (FGFR2) is a cell-surface receptor tyrosine kinase participating in physiological processes such as proliferation, differentiation and survival through signalization pathways for tissue repair. This receptor was described to be largely overexpressed in several solid tumor cancer types such as gastric cancer, colorectal or triple-negative breast cancer. Recently, treatment with FGFR2-TTC conjugate associated with ataxia telangiectasia and Rad3-related inhibitor (ATRi) was reported. Combinations of ATRi and FGFR2-TTC were evaluated on several cancer cell lines from breast (MFM-223 and SUM52-PE), colorectal (NCI-H716), and gastric cancer (KATO-III and SNU-16) [301] (Figure 10).

### 9.4. Clinical Evaluation

Mesothelin (or MSLN) is a glycoprotein anchored to the surface of mesothelial cells, mainly localized in serous cavities of the body as pleura, peritoneum or pericardium. Whereas its expression is limited in normal conditions, it is overexpressed in many cancers including malignant mesothelioma, pancreatic cancer, ovarian cancer or lung and breast cancers. Recently, a MSLN-TTC (BAY2287411) developed from anetumab, a humanized anti-mesothelin mAb, was evaluated in several cancer models. In vitro study performed on 12 MSLN-positive cell lines showed a correlation between the level of surface expression and measured IC_50_. Further experiments on OVCAR-3 cells illustrated the mode of action of MSLN-TTC such as inducing DSBs (validated by detection of a phosphorylated form of H2A histone family member X), production of reactive oxygen species due to ionizing radiation, as well as G2-M cell-cycle arrest, all these phenomena leading to cytotoxicity. Biodistribution and efficacy studies were performed on cell-line derived xenograft (CDX) and patient-derived xenograft (PDX) models. Based on doses of 100, 250 and 500 kBq/kg, tumor uptake and growth response increased with the level of MSLN of studied cell lines. Fractionation of 500 kBq/kg into 2 × 250 or 4 × 125 kBq/kg showed a similar impact on tumor growth than single dose treatment, even if the response was slower due to the lower effective dose received by the tumor [302]. Combination of MSLN-TTC conjugate with DNA damage response inhibitors was recently reported, the idea being to prevent activation of DNA repair mechanisms in order to maximize damages induced by MSLN-TTC leading to cell cycle arrest. Even if the four inhibitors demonstrated a synergistic effect on several cancer cell lines, impact was more pronounced for ATRi and polyadenosine diphosphate ribose polymerase inhibitor (PARPi). Consequently, treatment strategy with both inhibitors was evaluated in vivo on mice with OVCAR-3 xenografts since a strongest synergy was measured for this cell line. Association of ATRi (40 mg/kg) + 100 kBq/kg gave a similar response on growth inhibition than a single dose of 250 kBq/kg, when effect of PARPi (50 mg/kg) + 100 kBq/kg was not significantly different than single injection of 100 kBq/kg. This can be explained by a different mode of action of PARP that is related to single-strand-breaks repair whereas ATR acts directly on DSBs. Consequently, results are not surprising since α-particles are known to cause mainly direct DSBs. Then, in further experiments on OVCAR-8 cell line (lower level expression of MSLN receptors and more rapid growth rate), similar moderate percentage of inhibition were measured for monotherapy with 3 × 200 kBq/kg of MSLN-TTC or ATRi (40 mg/kg). On the other hand, combination of both treatments resulted in a clear synergistic effect on growth inhibition [303].

The HER2 receptor, previously presented as target of ^227^Th-trastuzumab, was also recently studied with the new generation of HER2-TTC. As mentioned before, after being submitted to radiations, cells activate DNA repair mechanisms and especially enzymes binding to single-strand DNA in order to restore double-strand DNA as poly ADP ribose polymerase 1 (PARP-1) and PARP-2. Investigations were conducted with HER2-TTC to promote DNA damages in combination with PARP inhibitors (PARPi) to limit cellular response. The HER2-TTC/PARPi combination was evaluated on colorectal adenocarcinoma HER-2 positive cell line DLD-1 parental and DLD-1 BRCA2 −/− with an inactivation of BRCA2, a gene involved in DNA DSBs repair. Deficient BRCA2 −/− cells appeared to be more sensitive to monotherapy with PARPi (olaparib) or HER2-TTC in comparison with DLD-1 parental cell line. This tendency was confirmed in tests with combined treatment in which synergic effect was measured for DLD-1 BRCA −/ cell line while only additive effect was determined for DLD-1. In vivo study after intravenous injection of 120, 300 or 600 kBq/kg of HER2-TTC illustrated a dose-dependent tumor growth inhibition in both cell lines. It has to be mentioned that effect of 600 kBq/kg was more pronounced in DLD-1 BRCA2 −/−, traducing a higher sensitivity to HER2-TTC. Monotherapy with PARPi (25 or 50 mg/kg) also led to an anti-tumor effect only on the DLD-1 BRCA2 −/− cell line. Similarly, combination of PARPi with 120 kBq/kg (that demonstrated a low effect as monotherapy) or 300 kBq/kg HER2-TTC appeared to be synergistic effect on tumor growth but not for parental DLD-1 cell line [304].

The last targeted thorium-227 conjugate reported was towards PSMA or more specifically, glutamate carboxypeptidase II (GPCII) regulated by folate hydrolase 1 (FOLH1) gene and specific of prostate cancer cells (including in mCRPC). The corresponding efficacy study from administration of 75, 150 or 300 kBq/kg (antibody amount fixed at 0.43 mg/kg) of PSMA-TTC (BAY2315497) demonstrated a significant antitumor effect. As a mimic of advanced stage of prostate cancers, further experiments were conducted on castration resistant models (MDA-PCa-2b, 22Rv1 and C4-2). Even if higher doses of PSMA-TTC (100, 250 and 500 kBq/kg) with lower antibody dose (0.14 mg/kg) were injected, a marked effect on growth inhibition was also observed and appeared as independent of the evolution of cancer cells. Others tests on PDX with ST1273, enzalutamide-resistant KUCaP-1 and LuCaP 86.2 models followed the same tendency with a clear effect on growth inhibition resulting in partial or even complete response after treatment with 500, 300 and 300 kBq/kg, respectively. Complementary experiments revealed that the total antibody dose injected (500 kBq/kg with 0.14, 0.75 or 5 mg/kg) had no influence on distributions (with 0.14 or 0.75 mg/kg) or induced a decrease in tumor uptake (with 5 mg/kg only). Simultaneously, treatment fractionation (4 × 125 kBq/kg or 2 × 250 kBq/kg) appeared to result in similar effects on tumor growth whatever the protocol used [305].

Bayer AG has a strong presence in the development of ^227^Th radioimmunoconjugates and is at the origin of all classified clinical trials. The first Phase I concerning evaluations of dose-escalation, tolerability and maximum tolerated dose of BAY1862864 (^227^Th-epratuzumab) in refractory CD22-positive non-Hodgkin lymphoma, started in November 2015. This study was complete in November 2019 and results have not been reported to date (NCT02581878). The promising preclinical data presented previously for the last three ^227^Th-RIC allowed them to achieve clinical research stage. Phase I recruitment opened in April 2018 and is still ongoing until the end of 2024 for BAY2287411 (or MSLN-TTC) investigated in patients with solid tumors expressing mesothelin (NCT03507452). For BAY2701439 (or HER2-TTC), developed to target cancers with HER2 expression as breast cancer or gastric cancer, a Phase I opened in November 2019 and is expected to end by 2026 (NCT04147819). Finally, Phase I for BAY2315497 (or PSMA-TTC) started in December 2018, in patients with mCRPC and should end in 2023 (NCT03724747).

In this part, we reported a global overview of the potential of ^227^Th other recent reviews dedicated to this radionuclide can provide additional information [306,307,308].

## 10. Conclusions

This review provides a detailed summary of most promising α-emitters describing each step of the investigations with α-emitters from the production, physico-chemical properties, preclinical studies to finish by the reported clinical evaluations.

To summarize, the development of ^225^Ac can be clearly noticed at all levels of the process leading to clinical applications. Despite some problems to supply the actual demand, alternative production routes would allow to improve its availability over the next few years. As in radiochemistry, an increasing number of chelating agents are being reported for stable coordination of ^225^Ac, that naturally facilitate the preparation of ^225^Ac-radioconjugates. At the moment, ^225^Ac seems to be ahead in comparison with other α-emitters. The interest of Big Pharma on ^225^Ac is also due to its 9.9 d half-life that allows for centralized production at the level of a continent. Its daughter ^213^Bi was the first α-emitter to reach clinical evaluations, and naturally, future production is directly linked to that of ^225^Ac. Nevertheless, chemistry of Bi is well known and ^213^Bi exhibit a limited but efficient range of chelating agent. ^213^Bi has been widely studied and showed promising results in preclinical studies even if its short half-life is a hindrance to further development. New production routes of ^225^Ac, in particular those where ^227^Ac is coproduced, are well adapted to ^225^Ac/^213^Bi generator which will allow a greater availability of this radionuclide. The analogue ^212^Bi was also a precursor among investigated α-emitters, but once again, the short half-life limited studies with pure ^212^Bi and the use of its parent ^212^Pb as an in vivo generator was globally preferred. The potential problems for accessibility to ^212^Pb are currently being solved, especially with the strategy of Orano Med (Bessines-sur-Gartempe, France), which decided to increase its production capacities with a larger stock of ^232^Th. The chemistry of ^212^Pb is also well documented and the few chelators that were reported are efficient for its chelation and the prevention of ^212^Bi release, facilitating the translation to preclinical and even clinical evaluations. Besides, the possible theranostic application with ^203^Pb isotopes is another argument supporting the development of ^212^Pb. From its physio-chemical properties, ^211^At often appears as one of the most promising α-emitters. Despite the fact that astatine chemistry in one of the less investigated, some alternatives allowed the development of methods resulting in several preclinical studies among which few were translated to clinical phase. Astatine availability has long been a limit to its development, nevertheless, this tendency is reversing and will have a promising impact all along ^211^At-radioconjugates conception. Currently, whether on international or European levels, interest for ^211^At is growing and research teams are structuring into three major networks: USA (DOE), Japan and Europe. In parallel, private companies are either making accelerators for alpha particle available or directly investing in ^211^At production. Unlike many other α-emitters, the availability of ^223^Ra was not an impediment to its study and conjugated to the use of a simple chemical form, it probably favored the interest for this radionuclide. In addition, the promising results noticed during preclinical and clinical phases in mCRPC cases have increased its potential for radiotherapy. Since 2010, publications on work with ^223^Ra really increased and represent now more than 75% of the clinical studies involving an α-emitter. However, at present, the difficulty of making stable compounds with ^223^Ra leads the community to move to ^227^Th. ^149^Tb is probably the α-emitter with the most challenging production method leading to a very limited availability. However, in terms of chemistry, suitable agents are described for lanthanide chelation and the resulting complexes demonstrated promising preclinical data. Besides, the possible association of ^149^Tb with other terbium radioisotopes exhibiting appropriate features for both imaging and therapy represents a non-negligible interest. Finally, despite a production process allowing a subsequent level of ^227^Th, its availability remains limited to few research groups in the world. Nevertheless, the chemistry of actinides is quite well understood and has resulted in the development of efficient chelating agents for ^227^Th. Studied in a wide range of pathologies, the resulting targeted ^227^Th-conjugates demonstrated a clear efficacy in preclinical investigations. Even if access to this radionuclide is difficult and that few ^227^Th-RIC were actually developed, most of them demonstrated promising results and reached clinical phase studies. ^227^Th may not be the most popular or the most developed of all α-emitters, but it is obvious that it shows a non-negligible potential.

In conclusion, all α-emitters have both their own advantages and disadvantages and none has demonstrated a great superiority compared to others. However, the recent work highlighted the increasing interest for ^225^Ac, ^211^At or ^213^Bi. Concerning ^212^Bi, its use was overtaken by ^212^Pb that is also emerging for TAT. Despite a clear impact on the development of TAT, ^223^Ra is well-known and now routinely used but may not be at the origin of important innovation in that domain. Other α-emitters as such ^149^Tb or ^227^Th also showed very promising results, but their availability remains limited, slowing their development. These α-emitters are all assets for the development of TAT and provide new therapeutic opportunities for the future.

## Figures and Tables

**Table 1:** Reported ^225^Ac preclinical studies involving an identified pathology model (non-exhaustive list).

Preclinical Model	Molecular Target	Targeting Vector	Chelating Agent	Investigations	References
Human ovarian carcinoma HER2-positive (SKOV-3 cell line)	HER2	trastuzumab	py4pa	Stability study, in vitro and biodistribution	[39]
Human breast cancer cell lines SUM-225 and MDA-MB-231			DOTA	In vitro, biodistribution (comparison with ^111^In-DTPA-trastuzumab), optical imaging and therapy study.	[46]
Human HER2-positive cell lines SKOV-3 (ovarian cancer) and MDA-MB-231 (breast cancer)		2Rs15d (nanobody)	DOTA	In vitro and biodistribution In vitro, therapy study, dosimetry and toxicity	[47,48]
U87mg human glioblastoma tumor cells	α_v_β_3_ integrin	c[RGDfK]	DOTA	Biodistribution, optical imaging and therapy study	[49]
Human glioblastoma cell line U251	Interleukin-13 receptor alpha 2 (IL13RA2)	PepL1	DOTA	Bioluminescent imaging, therapy study (comparison with ^64^Cu-PepL1)	[50]
NT2.5 mammary tumor cell line	Programmed cell death ligand 1 (PD-1)	Anti-mouse PD-L1 (anti-PD-L1-BC)	DOTA	Biodistribution (comparison with ^111^In-DTPA- anti-PD-L1-BC), imaging, dosimetry	[51]
Human prostatic carcinoma cells LNCaP	PSMA	RPS-070	Macropa	In vitro and biodistribution	[40]
		RPS-074	Macropa	In vitro, biodistribution, therapy study and dosimetry	[52]
Human pancreatic cell line BxPC3	Carbohydrate antigen 19.9	Human antibody 5B1	DOTA	Biodistribution, luminescence imaging, therapy studies (pre-targeting or conventional) and toxicity	[33,34]
Mammary carcinoma cell lines MFM-223 and BT-474	Human kallikrein peptidase 2 (hK2)	Humanized monoclonal antibody hu11B6	DOTA	In vitro, biodistribution and therapeutic study	[53]
Triple-negative breast cancer model SUM149T	Insulin growth factor receptor (IGF-1)	Human monoclonal antibody cixutumumab (IMC-A12)	DOTA	In vitro, imaging, biodistribution (comparison with ^111^In-cixutumumab) and efficacy study	[54]
Malignant melanoma cell line B16F10	Melanocortin-1 receptor (MC1R)	CycMSH	DOTA	Stability study and biodistribution	[25]
		αMSH	Crown	Stability study and biodistribution	[41]
Human cutaneous melanoma cells A375 and A375/MC1R and human uveal melanoma cells MEL270		MC1RL	DOTA	In vitro, pharmacokinetic, biodistribution, therapy study and dosimetry	[55]
Human cutaneous melanoma cells A375 and A375/MC1R			DOTA	Biodistribution, Pharmacokinetic, therapy study and toxicity	[56]
Malignant melanoma cell line B16F10	Very late 4 (VLA-4 or integrin α_v_β_1_)	Anti-mouse/human VLA-4 (CD49d)	DOTA	In vitro, biodistribution, imaging dosimetry and therapeutic efficacy	[57]
Human embryonic kidney epithelial cells HEK-293T and HEK-293T-Hx16	Delta like 3 protein (DLL3)	Humanized site-specific antibodies N149, SC16.56	DOTA	In vitro, biodistribution and efficacy study (comparison with ^177^Lu-DOTA-MMA)	[58]
Colorectal cancer (SW1222), breast cancer (BT-474) or neuroblastoma (IMR32)	GPA33 antigen	Humanized A33 and C825 (huA33-C825)	DO3A	Biodistribution (comparison with ^111^In-Pr, imaging) therapy study and toxicity (Pretargeted radioimmunotherapy)	[59]
Human pancreatic cell lines PANC-1 and MIA PaCa-2	Fibroblast activation protein (FAP)	FAP inhibitor (FAPI-04)	DOTA	In vitro, biodistribution and efficacy study	[60]
Human squamous carcinoma A431 cell line	Cholecystokinin B receptor (CCKBR)	PP-F11N	DOTA	In vitro, biodistribution and therapy study	[61]
Hepatoblastoma cell line HepG2 and squamous carcinoma A431 (GPC3+)	Glypican-3 (GPC3)	Codrituzumab (GC33)	Macropa	In vitro, biodistribution, therapy study and toxicity	[62]

**Table 2:** Preclinical studies involving an identified pathology model (non-exhaustive list).

Preclinical Model	Molecular Target	Targeting Vector	Prosthetic Groups	Investigations	References
Human ovarian carcinoma HER2-positive (SKOV-3 cell line)	HER2	2Rs15d (nanobody)	[^211^At]SAGMB [^211^At]SAB *^a^* [^211^At]MSB *^b^*	In vitro, dosimetry, and biodistribution	[121]
Human gastric cancer cell line NCI-N87		trastuzumab	[^211^At]SAB *^a^*	In vitro, biodistribution and therapy	[122]
BT474M1 human breast carcinoma cells		sdAb 5F7 (single-domain antibody fragments)	[^211^At]SAGMB *iso*-[^211^At]SAGMB	In vitro and biodistribution	[123]
Human papillary thyroid carcinoma K1 cells	Sodium-iodide symporter (NIS)	-	-	In vitro, toxicity, biodistribution and therapy	[124]
MDA-MB-231 and EMT-6 breast cancer cells	sigma-2 receptor	[^211^At]MM3	SE_Ar_	In vitro, biodistribution and dosimetry	[125]
Osteosarcoma (SaOS2, U2OS), colon cancer (HCT116, HCT116 p53^−/−^), human cervical cancer (HeLa), glioma (T98G) cell lines	Class I chain-related protein A and B (MICA/B)	Anti MICA/B	[^211^At]SAB *^a^*	In vitro, biodistribution and toxicity	[126]
Human non-small cell lung cancer (NSCLC) cell line A549	Somatostatin receptor-2 (SSTR2)	Octreotide	[^211^At] [^211^At]-SPC	In vitro, biodistribution and therapy	[127]
Human synovial sarcoma cell line SYO-1	Frizzled homolog 10 (FZD10)	OTSA101 (anti-FZD10)	[^211^At]SAB *^a^*	Biodistribution, therapy and dosimetry	[128]
PC12 rat pheochromocytoma cells	Norepinephrine transporter system	[^211^At]MABG	SE_Ar_	In vitro, Biodistribution and therapy	[129]
NCI-H929 multiple myeloma cell line	CD38	OKT10	B10-NCS	In vitro, Biodistribution and therapy	[130]
Neuroblastoma cell line IMR-05	PARP-1	[^211^At]MM4	SE_Ar_	In vitro, Biodistribution and therapy	[131]
Human (U87MG, U87MG-IDH1, U118MG, LN18 and mouse (GL26) glioblastoma cell lines				In vitro, toxicity and therapy	[132]
U-87MG glioblastoma cell line	α_v_β_3_ integrin	c[RGDfK]	SE_Ar_	In vitro and Biodistribution	[133]
Castration-resistant prostate cancer cell line PC3 transfected to express PSCA (PC3-PSCA)	Prostate stem cells antigen (PSCA)	A11 (minibody)	[^211^At]SAB *^a^*	Biodistribution, toxicity and therapy	[134]
PC3 prostate cancer cell lines	Gastrin-releasing peptide receptors (GRPRs)	Bombesin	[^211^At]SAB	In vitro and Biodistribution	[135]
C6, U-87MG and GL261 glioma cell lines	L-type amino acid transporter-1 (LAT-1)	*L*-Phenylalanine (PA), α-methyl-*L*-phenylalanine (AAMP) or α-methyl-*L*-tyrosine (AAMT)	SN_Ar_	In vitro, Biodistribution and therapy	[136]
Human ovarian carcinoma SKOV-3 cell line			SE_Ar_	In vitro, Biodistribution and therapy	[137]
Human pancreatic (PANC-1) and murine melanoma metastatic (B16F10) cell lines			SE_Ar_	In vitro, Biodistribution and therapy	[138]
mGluR1 expressing B16F10 melanoma cells	Metabotropic glutamate receptor type 1 (mGluR1)	[^211^At]AITM	SE_Ar_	In vitro, Biodistribution and therapy	[139]
5T33 murine myeloma cell line	CD138	9E7.4 (Anti-mCD138 antibody)	[^211^At]SAB	In vitro, Biodistribution, therapy and dosimetry	[140]
Human AML cell line U937	CXCR4	CXCR4 mAb	[^211^At]SAB	In vitro, Biodistribution and dosimetry	[141]
Human ovarian cancer cell line NIH:OVCAR3	folate receptor alpha (FRα)	Farletuzumab (MORab003)	[^211^At]SAB *^a^*	In vitro, biodistribution, therapy and dosimetry	[142]
C4-2B human prostate cancer cells (LNCaP subline)	PSMA antigen	Lysine-urea-glutamate (LuG) moiety	*Closo*-decaborate(2-) derivative	In vitro and biodistribution	[143]

*^a^* One-step radiolabeling using N-succinimidyl-3-(trimethylstannyl)-benzoate (m-MeATE) [114]; *^b^* One-step radiolabeling using N-2-(maleimido)ethyl-3-(trimethylstannyl)benzamide (MSB) [115].

## Data Availability

Not Applicable.
